# IgG Antibodies to Cyclic Citrullinated Peptides Exhibit Profiles Specific in Terms of IgG Subclasses, Fc-Glycans and a Fab-Peptide Sequence

**DOI:** 10.1371/journal.pone.0113924

**Published:** 2014-11-26

**Authors:** Susanna L. Lundström, Cátia Fernandes-Cerqueira, A. Jimmy Ytterberg, Elena Ossipova, Aase H. Hensvold, Per-Johan Jakobsson, Vivianne Malmström, Anca I. Catrina, Lars Klareskog, Karin Lundberg, Roman A. Zubarev

**Affiliations:** 1 Division of Physiological Chemistry I, Department of Medical Biochemistry and Biophysics, Karolinska Institutet, Stockholm, Sweden; 2 Rheumatology Unit, Department of Medicine, Karolinska Institutet, Stockholm, Sweden; National Cancer Institute at Frederick, United States of America

## Abstract

The Fc-glycan profile of IgG_1_ anti-citrullinated peptide antibodies (ACPA) in rheumatoid arthritis (RA) patients has recently been reported to be different from non-ACPA IgG_1_, a phenomenon which likely plays a role in RA pathogenesis. Herein we investigate the Fc-glycosylation pattern of all ACPA-IgG isotypes and simultaneously investigate in detail the IgG protein-chain sequence repertoire. IgG from serum or plasma (S/P, n = 14) and synovial fluid (SF, n = 4) from 18 ACPA-positive RA-patients was enriched using Protein G columns followed by ACPA-purification on cyclic citrullinated peptide-2 (CCP2)-coupled columns. Paired ACPA (anti-CCP2 eluted IgG) and IgG flow through (FT) fractions were analyzed by LC-MS/MS-proteomics. IgG peptides, isotypes and corresponding Fc-glycopeptides were quantified and interrogated using uni- and multivariate statistics. The Fc-glycans from the IgG_4_ peptide EEQ**F**NST**Y**R was validated using protein A column purification. Relative to FT-IgG_4_, the ACPA-IgG_4_ Fc-glycan-profile contained lower amounts (p = 0.002) of the agalacto and asialylated core-fucosylated biantennary form (FA2) and higher content (p = 0.001) of sialylated glycans. Novel differences in the Fc-glycan-profile of ACPA-IgG_1_ compared to FT-IgG_1_ were observed in the distribution of bisected forms (n = 5, p = 0.0001, decrease) and mono-antennnary forms (n = 3, p = 0.02, increase). Our study also confirmed higher abundance of FA2 (p = 0.002) and lower abundance of afucosylated forms (n = 4, p = 0.001) in ACPA-IgG_1_ relative to FT-IgG_1_ as well as lower content of IgG_2_ (p = 0.0000001) and elevated content of IgG_4_ (p = 0.004) in ACPA compared to FT. One λ-variable peptide sequence was significantly increased in ACPA (p = 0.0001). In conclusion, the Fc-glycan profile of both ACPA-IgG_1_ and ACPA-IgG_4_ are distinct. Given that IgG_1_ and IgG_4_ have different Fc-receptor and complement binding affinities, this phenomenon likely affects ACPA effector- and immune-regulatory functions in an IgG isotype-specific manner. These findings further highlight the importance of antibody characterization in relation to functional *in vivo* and *in vitro* studies.

## Introduction

Rheumatoid arthritis (RA) is a common chronic autoimmune disease characterized by joint inflammation and subsequent cartilage and bone destruction [1]–[4]. The presence of anti-citrullinated peptide antibodies (ACPA) in patients with more severe disease progression and in asymptomatic individuals years prior to disease onset, suggests that these autoantibodies play an important role in RA pathogenesis [1], [5], [6]. It has recently been demonstrated that the IgG-Fc-region in ACPA has distinct features, both in terms of the distribution of IgG subclasses and IgG_1_-Fc-glycosylation pattern [7]–[10]. The ACPA-IgG_1_-Fc region contains more truncated forms compared to the total IgG_1_ pool [7], which becomes more pronounced following onset of the disease [10]. In addition, IgG_1_ and IgG_4_ have been reported to be the predominant subclasses of antibodies that react with cyclic citrullinated peptides (CCP), citrullinated vimentin and citrullinated fibrinogen [8], [9]. These features can influence the affinity of IgGs to Fc-receptors and complement, and thereby modulate their activity of effector functions and regulatory pathways[11]–[14].

It is possible to use LC-MS/MS proteomics methodologies to investigate features in the IgG_1–4_ repertoire, both on the peptide sequence level and for the Fc-glycosylation pattern. The protein sequence region containing the N-linked Fc-glycan can be characterized according to IgG subclasses after trypsin digestion that produces well-known peptides; IgG_1_: EEQ**Y**NST**Y**R [P01857], IgG_2_: EEQ**F**NST**F**R [P01859], IgG_3_: EEQ**Y**NST**F**R [P01860] and IgG_4_: EEQ**F**NST**Y**R [P01861], (accession numbers correspond to UniProt IDs) [15], [16]. Known polymorphisms in IgG_3_ also result in EEQ**F**NST**F**R [17], [18]. This variant is frequently found in individuals of European descent [17], [18].

**Figure 1:**
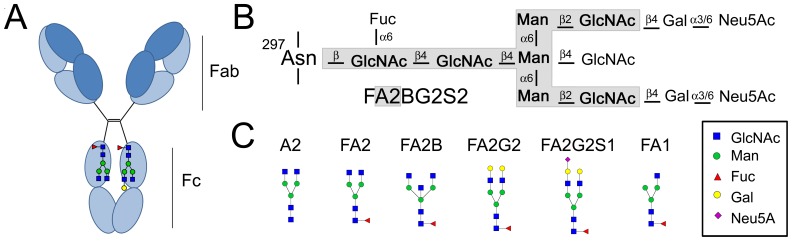
Schematic IgG protein and Fc-glycan structures. (**A**) The IgG protein. Locations of the Fab- and Fc- regions as well as of the Fc-glycans are indicated. (**B**) Schematic picture of N-linked biantennary oligosaccharide heterogeneity. Sugar identities, linkage positions and anomeric configurations are indicated. Nomenclature is given as described by Royle *et al* [19]. The core heptasaccharide moiety (A2) is gray shaded in contrast to the outer core variable units. F at the start of the abbreviation (FA2) indicates a fucose (Fuc) linked to the inner *N*-acetyl-glucosamine (GlcNAc). B indicates a bisecting GlcNAc linked to the middle mannose (Man), Gn indicates n (number of) galactoses (Gal) linked to antenna and Sn indicates n sialic acids (N-acetyl neuraminic acid, Neu5Ac) linked to Gal. If the glycan is monoantennary, (i.e. β-GlucNAc(1→2)-α-Man-(1→3)-[α-Man(1→6)]-β-Man-(1→4)-β-GlcNAc-(1→4)-β-GlcNAc-(1→Asn_297_) or β-GlucNAc(1→2)-α-Man-(1→6)-[α-Man(1→3)]-β-Man-(1→4)-β-GlcNAc-(1→4)-β-GlcNAc-(1→Asn_297_), respectively), it is referred to as A1. (**C**) Examples of the structural diversity.

As shown in Figure 1, the IgG-Fc attached oligosaccharide comprises a core biantennary heptasaccharide moiety ([A2]; nomenclature is according to Royle *et al*. [19]). If the glycan is mono-antennary, it is referred to as A1 [19]. The first sugar unit (an *N*-acetyl-glucosamine) is normally core fucosylated (e.g. FA2, FA1) [20]. An absence of this fucose may affect antibody effector functions. For example, the afucosylated IgG_1_ variant has 50- to 100- fold higher affinity to FcγRIIIa and the increased affinity is associated with an enhanced antibody-dependent cellular cytotoxicity (ADCC) [14], [21]. Similarly, if the biantennary structure is bisected with an extra *N*-acetyl-glucosamine (FA2B), it can also increase the affinity to FcγRIII and display more potent ADCC [22]. The outer glucosamine units can be elongated with galactoses (FA2Gn, n = 1 or 2) and the galactoses can be further extended with sialic acids (FA2GnSn, n = 1 or 2). The presence of terminal sialic acid on the glycan reduces Fc-receptor affinity 10-fold and, α-2,6-sialylation on the Fc-glycan can actively suppress inflammation via binding to the Specific Intercellular adhesion molecule-3-Grabbing Non-integrin (SIGN) receptors [14], [23].

Some aspects of Fc-glycosylation of IgG and ACPA from RA patients, and in particular of ACPAs from the IgG_1_ subclass, have been described previously [7], [10], [24]–[27]. However, there is no information available that describes the glycosylation patterns of ACPAs which takes into account both detailed glycosylation patterns of all IgG subclasses as well as the quantitative distribution of these subclasses in serum/plasma (S/P) and synovial fluid (SF) of RA patients. In the present study, we have used one single method, proteomic-type LC-MS/MS [28], to simultaneously investigate differences both in the protein/peptide abundances and in IgG_1–4_-Fc glycan structures in ACPA-IgG and in non-ACPA-IgG derived from serum/plasma and synovial fluid samples from ACPA positive RA patients.

## Materials and Methods

### Subjects and samples

Serum (n = 8), plasma (n = 7) and synovial fluid ([SF], n = 4) were obtained from 18 anti-CCP2 positive patients diagnosed with RA according to the ACR/EULAR 2010 criteria (Serum/Plasma [S/P]: 22–77 years, 6 females; SF: 42–69 years, 2 females) (Supplementary Table S1). SF from three of the patients was sampled at two occasions, with one year between sampling dates. Nine of the patients were sampled following short symptom duration (0.5–4 years) and nine of the patients were sampled following long symptom duration (16–47 years), (Table S1) [29]. Patients were attending the Rheumatology Clinic at Karolinska University Hospital, and were receiving health care and treatment according to clinical guidelines and practice. All participants gave their oral consent as specified in the ethical approval (Case number 2006-476-31/4, Stockholm Regional Ethics Committee), and in line with Swedish law. Patient’s consent was documented in the medical records by the respective treating physician. This was done after the patient had received information about the study and after approving participation in the study.

### Protein G and CCP2 column purification

ACPA (anti-CCP2 reactive IgGs) were obtained as recently described [30]. Briefly, SF (15–25 mL) samples were treated with hyaluronidase and centrifuged at 3000 g for 5 min. Supernatant proteins were precipitated with saturated ammonium sulphate and dialyzed against PBS. Plasma and serum (10–20 mL) samples were centrifuged at 3000 g for 5 min and diluted 1∶5 (v/v) in PBS. IgGs from SF and diluted S/P samples were purified on HiTrap Protein G HP columns (GE Healthcare, Sweden), according to the manufacturer’s instructions. Eluted IgGs were dialyzed against PBS and applied to CCP2 affinity columns (kindly provided by Euro-Diagnostica, Sweden). ACPAs were eluted using 0.1 M glycine–HCl buffer (pH 2.7) and pH was immediately adjusted to 7.4 with 1 M Tris (pH 9). The unbound IgG fraction, i.e. the “flow through”, (FT), was also collected. Both ACPA and FT fractions were concentrated and buffer exchanged to PBS using 10 kDa Microsep UF Centrifugal Device (Pall Life Science, USA). Recovery degree and purity of total ACPAs were assessed by measuring anti-CCP2 reactivity (Immunoscan CCPlus assay, Euro-Diagnostica, Sweden) and by SDS-PAGE/Coomassie Brilliant Blue staining.

### Protein A column purification

Protein A HP Spin Trap affinity columns (GE Healthcare, Sweden) were used in order to separate IgG_3_ from IgG_1_, IgG_2_ and IgG_4_ in both ACPA and FT samples previously purified from plasma, sera and SF. Due to limited sample material, pooled samples were used as well as individual samples from subjects 9, 11, 15 and 16 (Table S1). ACPA and FT samples were loaded on pre-equilibrated (20 mM sodium phosphate buffer, pH 7.4) protein A columns and incubated for 4 min with gentle mixing. IgG_3_ was collected by 30 s centrifugation at 75 g and the columns were washed two times with equilibration buffer (via centrifugation, 30 s at 75 g). Subsequently, IgG_1_, IgG_2_ and IgG_4_ were jointly collected by eluting protein A columns with 0.1 M Glycin-HCL, pH 2.7. Eluates were collected in tubes containing pH neutralizing buffer (1 M Tris-HCL, pH 9). Samples were directly buffer-exchanged to PBS using 10 kDa Nanosep UF Centrifugal Device (Pall Life Science, Port Washington, NY).

### Sample preparations and liquid chromatography - mass spectrometry analysis

Prior to LC-MS/MS analysis, IgG samples (5 µg/sample) were digested by trypsin as previously described (29, 30). Briefly, samples were reduced with 20 mM dithiothreitol for 30 min at 56°C and alkylated with 66 mM iodoacetamide for 30 min in darkness. Trypsin was added (1∶50; enzyme:protein) and digestion was performed at 37°C overnight. Peptides were desalted using C18 StageTip (Thermo Fisher Scientific, Waltham, MA), dried using SpeedVac and resuspended in 0.1% formic acid and 0.5% acetonitrile solution. Samples were kept at 10°C and injected on the column in 5 µL aliquots containing 0.3 µg of digest.

Glycosidase treatment was performed over night at 37°C on trypsin digest using 2.5 mU of 1) α(2–3,6,8,9)-Sialidase A and 2) α(2–3,6,8,9)-Sialidase A and β(1–3,4)-Galactosidase (ProZyme, Hayward, CA) dissolved in 10 µL of the corresponding X10 buffers provided by the manufacturer.

Samples were analyzed in triplicates in a randomized order using reversed phase liquid chromatography (LC) system (Easy-nLC, Proxeon, Thermo Fisher Scientific) connected to a hybrid LTQ Orbitrap Velos ETD mass spectrometer (Thermo Fisher Scientific, Waltham, MA) operating in positive ion mode. The survey mass spectrum covering the range of m/z 300–2000 was obtained with a resolution of 60,000 at m/z 400. Following each MS scan, top five most abundant precursor ions were selected for MS/MS with collision induced dissociation (CID) and electron transfer dissociation (ETD). The instrument was calibrated externally using internal lock mass calibration on m/z 429.088735 and 445.120025. LC-separation of the peptides and glycopeptides were performed on a 10 cm long fused silica tip column (SilicaTips New Objective Inc.) packed in house with 3 µm C18-AQ ReproSil-Pur (Dr. Maisch GmbH, Germany). The chromatographic separation was achieved using a water (A) and acetonitrile (B) solvent system both containing 0.1% formic acid. The gradient was set up as following: 3−35% (B) in 35 min, 36−95% (B) in 5 min, 95% (B) for 8 min and 3% (B) for 10 min. The flow rate was set at 300 nl/min.

### Protein identification and quantification

The MS/MS spectra were extracted from.raw files into.mgf files using in-house written RAW_to_MGF software [31]. Mascot (Matrix Science) search engine v.2.3.02 was used for protein identification with a concatenated version of the SwissProt protein sequence database (April, 2013, 20242 entries). Peptide mass error tolerance was set at 10 ppm, MS/MS fragment mass accuracy at 0.5 Da and tryptic digestion was set with a maximum of two missed cleavages. Carbamidomethylation of cysteine was used as a fixed modification, while the variable modifications were asparagine and glutamine deamidation, methionine oxidation as well as N-glycosylation (HexNAc[m]dHex[n]Hex[o]; m, n and o are the number of *N*-acetyl-hexoseamines, deoxyhexoses and hexoses, respectively). Peptide and protein quantification was performed with in-house written software Quanti [31].

### Glycopeptide identification and quantification

IgG-glycopeptide amino acid sequences and glycoforms were characterized as previously described [28]. Briefly, IgG Fc-glycopeptides were identified in LC-MS/MS datasets by their characteristic retention times and accurate monoisotopic masses (within <10 ppm from the theoretical values) of doubly and triply charged ions (IgG_1_: EEQ**Y**NST**Y**R, IgG_2_ or IgG_3_ [IgG_2/3_]: EEQ**F**NST**F**R and IgG_4_ (or IgG_3_) [IgG_4/(3)_]: EEQ**F**NST**Y**R (or EEQ**Y**NST**F**R)) as well as of triply and quadruply charged ions (IgG_1_: TKPREEQ**Y**NST**Y**R, IgG_2/3_: TKPREEQ**F**NST**F**R and IgG_4/(3)_: TKPREEQ**F**NST**Y**R (or TKPREEQ**Y**NST**F**R)), Table S2. For additional validation of glycopeptide identities, retention times of glycopeptides from the IgG standard (Sigma Aldrich, St Louis, MO, Table S3) as well as MS/MS and deglycosylated peptides obtained by glycosidase treatments of ACPA and FT were used [28]. The close sequence homology of EEQ**F**NST**Y**R (IgG_4_) and EEQ**Y**NST**F**R (IgG_3_) around Asn_297_ results in identical monoisotopic masses and overlapping retention times of the corresponding glycopeptides (Figure S1). Therefore, these glycopeptides cannot *per se* be differentiated by LC-MS/MS. However, IgG_3_: EEQ**F**NST**F**R is more frequently found in individuals of European descent [17], [18]. Via protein A column separation of IgG_3_ and IgG_4_ with subsequent LC-MS/MS analysis (Figure S1), this information was confirmed. Thus, EEQ**F**NST**F**R glycopeptides are referred to as IgG_2/3_ and EEQ**F**NST**Y**R (or EEQ**Y**NST**F**R) glycopeptides are referred to as IgG_4/(3)_.

Quantification of glycoforms was performed in a label-free manner using Quanti [28], [31]. Glycopeptide ion abundances were integrated over respective chromatographic monoisotopic ion peaks (<10 ppm from the theoretical values) at the charged states described above and within a ±1.5 min interval around the expected retention times. Results were validated by manual qualitative and quantitative investigation of the.raw files. Glycoform abundances were normalized to total content (100%) of Fc-glycosylated IgG_1_ peptides, total content (100%) of Fc-glycosylated IgG_2/3_ peptides and total content (100%) of Fc-glycosylated IgG_4/(3)_ peptides, respectively.

### Statistical analysis

Univariate statistical analysis was performed using paired two-tailed Student’s t-test on matched FT *versus* ACPA data using mean values of the triplicate measurements for each individual (Table S3). Both serum and plasma enriched FT and ACPA fractions obtained from the same individual (subject 9, Table S1) were used to validate that the IgG profile was not affected by the matrix origin. The validation confirmed that the correlation between the measured glycan and protein levels in the serum and plasma generally was very good (details are given in Table S1). The mean of the combined plasma and serum measurements for subject 9 was used when comparing ACPA and FT statistically. As described above, the study included SF samples from three individuals that were tested at two occasions (one year between sampling dates). As shown in Figure S2 the samples show intra-individual similarities but are distinct in terms of FT and ACPA specificity. To avoid skewed statistical results, the mean values for the samples were used. Principal component analysis (PCA) and orthogonal projections to latent structures – discriminate analysis (OPLS-DA) were performed using SIMCA 13.0 (Umetrics, Umeå, Sweden) following log transformation, mean centering and UV scaling. Model performance was reported as cumulative correlation coefficients for the model (R^2^X[cum]) and predictive performance based on seven-fold cross validation calculations (Q^2^[cum]).

## Results

### IgG subclass and protein/peptide distribution in ACPA versus FT

**Figure 2:**
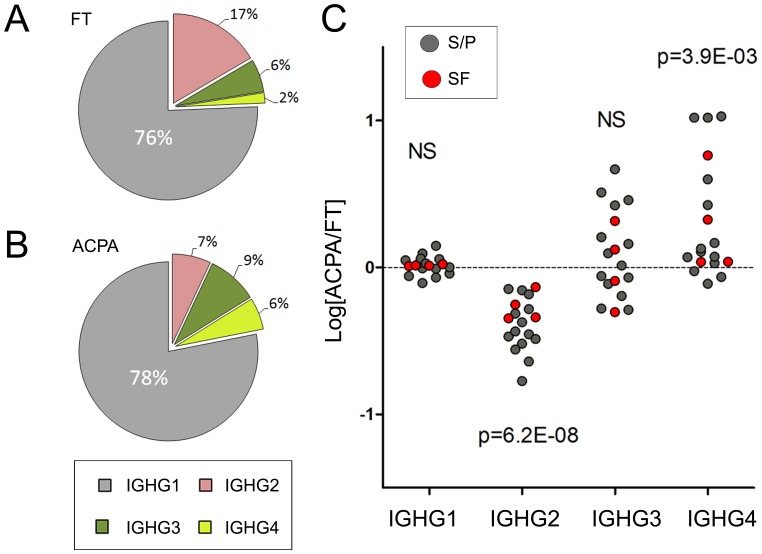
Composition (%) of IgG heavy chain isotypes. (**A**) FT isotype composition. (**B**) ACPA isotype composition. (**C**) Log_10_ fold change of the intra-individual ACPA/FT-ratio. Values ≥0 (dashed line) indicate an increase in ACPA. Given p-values (comparing FT and ACPA) were obtained with paired t-test. NS: Not Significant.

When comparing the relative abundances (%) of the heavy chains from IgG_1–4_, (IGHG1, IGHG2, IGHG3 and IGHG4), the most prominent IgG type was IgG_1_ in all samples, (FT: 76%±10%, ACPA: 78%±11%). The abundance of IgG_4_ was significantly higher (p = 0.004) in ACPA (6%±8%) compared to FT (2%±1%). In contrast, the abundance of IgG_2_ was significantly lower (p = 0.0000001) in the ACPA fractions (7%±3%) compared to the FT fractions (17%±8%). An overview of the IgG type distributions and intra-individual distribution change in FT and ACPA samples of SF or S/P is shown in Figure 2.

**Figure 3:**
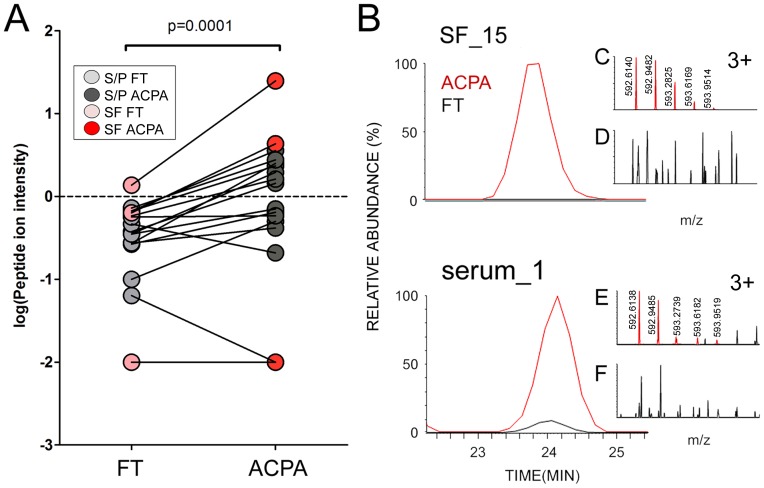
Differences between ACPA and FT in variable λ-chain peptide DFMLTQPHSVSESPGK. (**A**) Intra-individual differences between FT and ACPA. Given p-value was obtained with paired t-test. (**B**) Ion chromatograms of the peptide ion precursor (ACPA: red, FT: black) from a SF-IgG sample (upper panel) as well as from a serum-IgG sample (lower panel). (**C, E**) Mass spectra from ACPA from each patient, with the precursor ion isotopic pattern marked out in red. (**D, F**) The corresponding FT mass spectra for each individual, the precursor is weak/not identified.

We also observed differences in the abundance of heavy and light chain variants between the ACPA and FT fractions. Significantly elevated levels of a peptide previously found in the variable region of λ-chains LV603 and LV601 were found in the ACPA fractions (p = 0.0001, Figure 3). The sequence was identified as DFMLTQPHSVSESPGK via MS/MS with a score of 72 in SF (subject 15) and a score of 66 in S/P (subject 1), Figure S3. Additionally, significantly lower levels (p<0.04) of two λ-chains (LV301 and LV302), three κ-chains (KV119, KV106 and KV204), and one heavy chain (HV304), were observed in the ACPA fractions, Table S4. For identified peptide sequences, see Table S5.

The IgG heavy and light chains contributed to approximately 99.9% of all detected proteins in the FT fractions and 99.0% of all proteins in the ACPA fractions. Additionally, traces of IgM, IgA, complement 1 q (C1q) and CD5 antigen like protein (CD5L), could be detected (Table S5). All four of these proteins were observed with significantly higher abundances in the ACPA fractions compared to the FT fractions (Table S4).

### Significant intra-individual variations in Fc-glycans of ACPA-IgG_1_ compared to FT-IgG_1_

**Figure 4:**
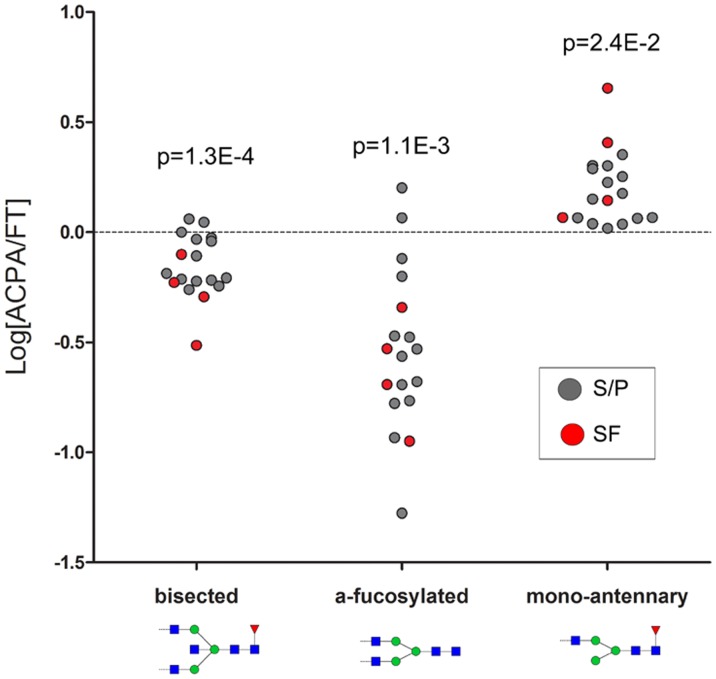
Log_10_ fold intra-individual ratio [ACPA/FT] of the sum of IgG_1_ bisected forms (n = 5), sum of IgG_1_ afucosylated forms (n = 4) and sum of IgG_1_ mono-antennary forms (n = 3). Values ≥0 (dashed line) indicate an intra-individually increased amount in ACPA. Given p-values (comparing FT and ACPA) were obtained with paired t-test. See Figure S4 for FT and ACPA differences in direct values.

**Figure 5:**
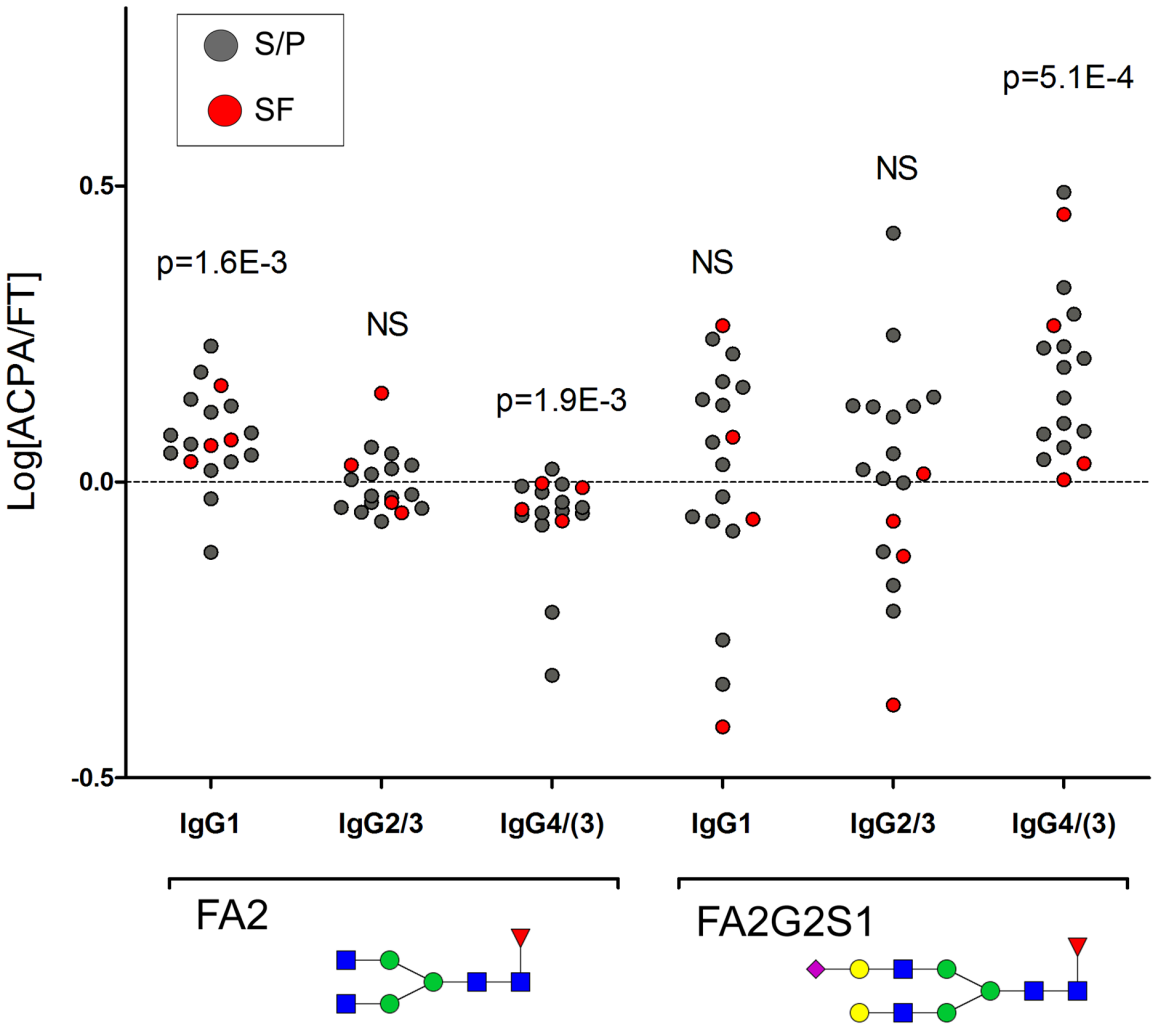
Log_10_ fold difference of FA2 and FA2G2S1 comparing the ACPA/FT ratio for the different IgG Fc-glycopeptide types. Values ≥0 (dashed line) indicate an intra-individually increased amount in ACPA. P-values (comparing FT and ACPA for each subject) were obtained with paired t-test. NS: Not Significant. See Figure S5 for FT and ACPA differences in direct values.

A total of 19 glycans substituting Asn 297 of IgG_1_ were searched after in the LC-MS/MS data using Quanti (Table S2). With the exception of FA2BG2S2, all glycoforms were detected in quantifiable amounts (Table 1). Four out of five of the identified bisected glycans (FA2B, FA2BG1, FA2BG2 and FA2BG2S1) were observed at significantly lower levels (p<0.01) in the ACPA (Table 1). Similarly, the sum of all bisected glycans (n = 5) were significantly decreased (p = 0.0001) in the ACPA-IgG_1_ as shown in Table 1, Figure 4 and Figure S4. Notable are also the differences observed when comparing FT-IgG_1_ and ACPA-IgG_1_ distributions of the mono-antennary forms (i.e. FA1, FA1G1 and FA1G1S1). All three of these glycans were significantly elevated (p<0.04) in ACPA-IgG_1_, Table 1, Figure 4 and Figure S4. As previously reported, we could confirm a significant decrease in afucosylated ACPA-IgG_1_ glycans (n = 4, p = 0.001), and a significant increase (p = 0.002) in the main FA2 glycan (Table 1, Figure 4 and S4, Figure 5 and S5, respectively). With the exception of FA2G2S2 (p = 0.01, S/P only, Table 1), ACPA-IgG_1_ were not found to be significantly less sialylated compared to the FT-IgG_1_.

**Table 1:** IgG Fc glycans in serum or plasma (S/P) and synovial fluid (SF) samples of ACPA *versus* FT.

IgG Type	Glycan^a^	S/P^b^		SF^c^		p-value (paired)	
		FT^d^	ACPA^e^	FT	ACPA	S/P (n = 14)	S/P+SF (n = 18)
		AV^f^±STD^g^	AV±STD	AV±STD	AV±STD	FT/ACPA	FT/ACPA
1	FA1	0.5±0.2	0.7±0.5	1±1	3±3	**1.48E-02**	**4.12E-02**
	FA1G1	0.1±0.1	0.2±0.1	0.2±0.1	0.3±0.1	1.87E-01	**4.01E-02**
	FA1G1S1	0.05±0.04	0.1±0.1	0.1±0.1	0.7±0.7	**4.51E-04**	**2.65E-02**
	ΣA1-forms	0.6±0.3	1.0±0.6	2±1	4±3	**2.85E-03**	**2.36E-02**
	FA2	30±8	35±9	39±10	48±16	**1.14E-02**	**1.63E-03**
	FA2G1	28±5	30±3	26±3	23±8	2.84E-01	7.12E-01
	FA2G2	12±4	12±4	8±4	8±3	4.60E-01	5.80E-01
	FA2G1S1	2±1	2±0.4	2±0.8	2±1	5.06E-01	6.95E-01
	FA2G2S1	6±3	6±2	5±3	4±2	9.20E-01	8.56E-01
	FA2G2S2	0.1±0.04	0.05±0.03	0.1±0.04	0.1±0.04	**1.29E-02**	6.46E-02
	ΣS-forms	8±3	8±2	7±4	6±2	8.80E-01	8.90E-01
	FA2B	7±2	5±2	8±3	4±3	**1.18E-02**	**4.55E-04**
	FA2BG1	7±3	6±4	7±5	5±4	9.12E-02	**1.34E-02**
	FA2BG1S1	0.2±0.1	0.1±0.1	0.3±0.1	0.3±0.2	7.00E-02	5.81E-02
	FA2BG2	1±1	0.8±0.5	0.8±0.5	0.5±0.5	**8.95E-03**	**1.22E-02**
	FA2BG2S1	0.1±0.1	0.1±0.1	0.1±0.03	0.1±0.1	**1.82E-02**	**1.15E-02**
	ΣB-forms	15±5	12±6	16±7	10±8	**4.28E-03**	**1.26E-04**
	A2	2±1	0.6±0.8	0.7±0.4	0.2±0.1	**3.88E-02**	**1.51E-02**
	A2B	0.4±0.4	0.2±0.4	0.2±0.3	0.01±0.01	1.38E-01	5.36E-02
	A2G1	3±1	0.9±0.7	0.9±0.6	0.3±0.4	**1.98E-03**	**7.19E-04**
	A2G2	1±1	0.4±0.3	0.3±0.4	0.1±0.2	**7.71E-04**	**4.88E-04**
	ΣaF-forms	6±4	2±2	2±1	0.6±0.6	**3.54E-03**	**1.14E-03**
2/3	FA1	0.7±0.4	0.8±0.9	2±1	1±0.6	3.47E-01	6.75E-01
	FA1G1	0.05±0.04	0.1±0.1	0.2±0.2	0.2±0.1	2.85E-01	7.40E-01
	FA1G1S1	0.1±0.1	0.2±0.3	0.2±0.4	0.2±0.2	1.42E-01	3.20E-01
	ΣA1-forms	0.8±0.4	1±1	2±2	2±0.6	2.14E-01	5.30E-01
	FA2	38±9	38±10	45±12	44±8	3.60E-01	4.89E-01
	FA2G1	31±3	31±5	28±6	25±5	8.87E-01	3.56E-01
	FA2G2	9±4	9±4	9±5	7±4	8.47E-01	3.63E-01
	FA2G1S1	4±2	4±2	2±0.5	3±1	8.83E-01	5.52E-01
	FA2G2S1	5±3	6±4	4±1	3±2	8.05E-02	1.63E-01
	ΣS-forms	9±4	10±5	6±2	6±3	3.21E-01	2.28E-01
	FA2B	6±2	6±3	6±4	5±4	**4.33E-02**	**1.38E-02**
	FA2BG1	3±1	4±2	3±1	2±1	4.67E-01	7.38E-01
	FA2BG2	0.6±0.6	0.5±0.4	0.3±0.2	0.3±0.2	3.87E-01	3.67E-01
	ΣB-forms	10±4	10±4	9±5	8±5	2.82E-01	9.59E-02
	A2	1±1	1±1	0.4±0.4	0.2±0.1	3.27E-01	4.38E-01
4/(3)	FA1	0.4±0.3	0.5±0.2	1±1	2±2	1.34E-01	1.17E-01
	FA2	44±8	38±9	51±16	49±18	**5.69E-03**	**1.92E-03**
	FA2G1	19±5	22±7	19±5	19±5	1.41E-01	1.81E-01
	FA2G2	11±5	12±4	7±3	8±6	3.31E-01	1.77E-01
	FA2G1S1	7±5	8±4	2±2	5±4	2.84E-01	1.07E-01
	FA2G2S1	4±2	6±3	3±2	5±2	**1.52E-03**	**5.13E-04**
	ΣS-forms	10±5	13±5	5±3	10±5	**2.51E-03**	**6.00E-04**
	FA2B	10±4	9±5	12±6	8±5	1.48E-01	**1.90E-02**
	FA2BG1	4±3	4±3	4±4	4±4	4.28E-01	3.16E-01
	FA2BG2	0.4±0.4	1±1	0.7±0.7	0.6±0.9	5.30E-02	9.76E-02
	ΣB-forms	14±8	13±9	16±10	13±9	6.41E-01	2.03E-01

Relative distributions (%), and their respective standard deviations are indicated. In addition to the individual glycan species the sum of the mono-antennary (ΣA1), sum of sialylated (ΣS), sum of bisected (ΣB) and sum of afucosylated (ΣaF) forms are shown. P-values comparing FT and ACPA were obtained using paired t-test; significant p-values (p<5.0E-2) are bolded.

Abbreviations: ^a^Glycan acronyms are provided in Figure 1A, ^b^Serum or Plasma, ^c^Synovial Fluid, ^d^Flow Through, ^e^Anti-Citrullinated Peptide Antibody, ^f^Average; ^g^Standard Deviation.

### Significant intra-individual variations in Fc-glycans of ACPA-IgG_4/(3)_ compared to FT-IgG_4/(3)_

Nine glycopeptides N-linked to either Asn_297_ of IgG_4_ or (IgG_3_ with peptide sequence EEQ**Y**NST**F**R) were detected in quantifiable amounts, Table 1. In contrast to ACPA IgG_1_ which showed significantly elevated FA2 levels compared to FT, FA2 abundances in ACPA-IgG_4/(3)_ were significantly lower (p = 0.002, Table 1, Figure 5, Figure S5). However, note that this difference possibly generates more similar FA2 levels in ACPA-IgG_1_ and ACPA-IgG_4/(3)_. Thus, in S/P, the FA2 distributions in ACPA for IgG_1_ and IgG_4/(3)_ were 35%±9% vs 38%±9% respectively, compared to the FT levels at 30%±8% vs 44%±8%, respectively. A similar trend is observed for the SF FA2 distribution in FT and ACPA (Table 1). In addition to the change in distribution of the FA2 glycan, FA2G2S1 as well as the combined abundance of glycoforms FA2G1S1 and FA2G2S1, were significantly elevated in the ACPA-IgG_4/(3)_ fractions (p = 0.001 and p = 0.001), Table 1, Figure 5 and Figure S5.

In order to confirm that IgG_3,_ with peptide sequence EEQ**Y**NST**F**R, is a minor component of the IgG_4/(3)_ glycopeptide pool, ACPA and FT samples were further purified using a Protein A column, digested and reanalyzed by LC-MS/MS. Compared to IgG_2_ (20%±12%) and IgG_4_ (3%±2%), IgG_3_ is the main isotype that is measured in the protein A column FT, 72%±12%. This, combined with the distribution of glycopeptides EEQ**Y**NST**F**R (5%±9%) and EEQ**F**NST**F**R (95%±9%) in the protein A column FT, indicates that the majority of IgG_3_ contains the EEQ**F**NST**F**R sequence (Figure S1). The results, i.e. a decrease of FA2 and an increase in the sialylated forms in ACPA vs FT were consistent following protein A column analysis of IgG_4_ in the protein A column elutes.

### Significant intra-individual variations in Fc-glycans of ACPA-IgG_2/3_ compared to FT-IgG_2/3_

With the exception of FA2B, which was significantly decreased (p = 0.01) in the ACPA-IgG_2/3_, no other significant differences were observed.

### Differences between the S/P and SF glycan-profiles

We could confirm that similarly to what has previously been reported [7], the general trend in the SF IgG_1_ glycans indicated lower levels of sialylation and galactosylation and a higher abundance of FA2 (Table 1, Figure S6). Notably, the same trend was also observed in both the IgG_2/3_ and in the IgG_4/(3)_ glycan profile. No attempt was done to test for statistically significant differences in the SF profiles compared to the S/P profiles due to the low number of SF samples in the study (n = 4). All individual data is provided in Table S3.

### Differences in the ACPA and FT glycan-profiles according to disease duration

With the exception of FA2G1S1 from ACPA-IgG_1_ (S/P) that was significantly more abundant (p = 0.03) in patients with short disease duration, no significant differences were observed in either ACPA or FT when comparing patients with long and short disease duration.

### Multivariate statistical modeling to identify overall trends in the data set

**Figure 6:**
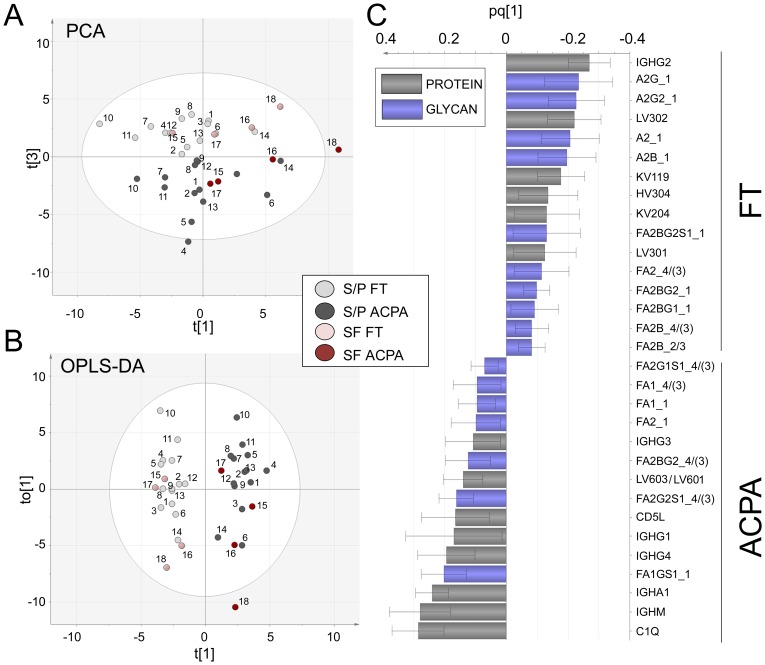
Multivariate modelling based on the Fc-glycan and protein/peptide correlations in the ACPA and FT sample set. Subjects are labeled according to Table S1 and colored according to FT (light gray [S/P]/light red [SF]) and ACPA (dark gray [S/P]/dark red [SF]). (**A**) PCA scores plot. FT and ACPA samples are separated in component t [3]. (**B**) OPLS-DA scores plot, the model was constructed to distinguish FT samples and ACPA samples and generated a strong (R^2^ = 0.93, Q^2^ = 0.85, CV ANOVA p-value = 2.3E-12) model, with distinct separation of FT (negative) an ACPA (positive) along the predictive X-axis t [1]. (**C**) Loading column plot of the predictive axis (pq [1]). Features with positive pq [1] values indicate positive ACPA correlation and features with negative pq [1] values indicate negative ACPA correlation. Only glycans (blue) and proteins (grey) correlating with 95% confidence are shown.

Multivariate statistical modeling integrating Fc-glycan (n = 39), IgG heavy and light chain (n = 25) as well as IgM, IgA, C1Q and CD5L data (Table S3) was performed in order to find overall trends in the data set. The PCA model constructed from the three first components (R^2^ = 0.46, Q^2^ = 0.16), showed a distinct separation between ACPA and FT samples in component t [3], (Figure 6A). Components t [1] and t [2] were mainly affected by inter-individual differences such as age and matrix type (SF or S/P). No distinct effects caused by disease- or symptom duration were observed.

An OPLS-DA model (R^2^ = 0.93, Q^2^ = 0.85, CV ANOVA p-value = 2.3E-12) was constructed in order to differentiate FT and ACPA samples according to the abundances of glycans and proteins. The high Q^2^-value (accounting for predictability) as well as the low model p-value, indicate that the model is robust. The separation of the predictive variance (ACPA or FT specific) from the orthogonal variance (unspecific noise caused by other inter-individual differences), greatly improves the interpretability of the OPLS-DA model compared to a PCA model. In the OPLS-DA scores plot (Figure 6B), the FT (−) and ACPA (+) samples are distinctly separated on the predictive X-axis (t [1]) while the non-ACPA/FT specific sample differences are indicated via the separation along the Y-axis (to [1]). The glycopeptides and proteins that distinguish the FT and ACPA samples with 95% confidence are shown in the loading column plot, Figure 6C. Proteins/Fc-glycans with positive pq [1] values correlate positively with ACPA, while those with negative pq [1] values correlate negatively with ACPA (i.e. they are positively FT-correlating). The OPLS-DA loading plot confirms and gives an overview of the core results obtained via the univariate statistical analyses (Table 1 and Table S4). Namely, that the main significantly elevated glycans in IgG_1_ (FA2, and the mono-antennary forms), as well as in IgG_4/(3)_ (sialylated glycoforms), have a strong correlation with the ACPA samples. Similarly, afucosylated forms and bisected forms from IgG_1_ and FA2 of IgG_4/(3)_ strongly anti-correlate with the ACPA samples. Notably, in addition to the IgG_4_ isotype and the variable chain sequence LV603/LV601, IgG_1_ and IgG_3_ also positively correlate with the ACPA samples with 95% confidence.

## Discussion

The present study uses a single analytical method (LC-MS/MS) to simultaneously investigate features on the level of peptide abundances and Fc-glyosylation of IgG_1–4_. Our data show distinct features of ACPA-IgG eluted from CCP2 affinity columns as compared to non-CCP2 reactive FT-IgGs from the same patients. The differences concern IgG subclasses, peptide-chain sequence repertoire as well as Fc-glycosylation profiles.

We have previously reported that the relative ACPA (anti-CCP IgG) distribution in the overall IgG pool of ACPA positive patients are approximately 1.5% in plasma and 2.2% in SF [30] and correspond to a median ACPA concentration of 1 µM in plasma and 0.4 µM in SF, respectively. Since IgG has a high occupancy of Fc-glycans *in vivo* [28], [32], the concentration of Fc-glycosylated IgG would be in a range proportional to these numbers. However, in terms of the distribution of the different glycoforms, it is important to take into account that (and as shown in Figure 1A), each IgG molecule can be substituted by two glycan’s and that differences in the combination of substituted glycotypes (FA2+FA2G2, FA2+FA2, e.g.) introduces an additional and potentially important level of complexity.

In line with previous findings [8], [9], we could demonstrate that IgG_4_ is significantly elevated (p = 0.004) in ACPA fractions as compared to FT fractions (Figure 2), and we provide further novel intra-individual data showing that ACPA-IgG_4_ Fc-glycans are significantly more sialylated (p = 0.001) and contain less amounts of FA2 (p = 0.002) compared to FT-IgG_4_ (Table 1, Figure 5). The differences in biological properties of IgG_4_ compared to IgG_1_ and IgG_3_ suggest that the effects of IgG_4_ ACPA may be different from the effects of ACPAs of the other subclasses. For example, IgG_4_ antibodies are poor inducers of complement and Fc-receptors [11], [12], [33], but have anti-inflammatory properties, including “Fab-arm exchange” [34], i.e. the ability to swap one heavy and light chain pair with another molecule, resulting in bi-specific antibodies. IgG_4_ antibodies also have the ability to target the Fc-region of other IgGs via its Fc-, rather than its Fab-region, thereby contributing to the clearance of IgG and IgG-bound material [35]–[37]. It could be hypothesized that the composition of Fc-glycans from ACPA-IgG_4_ may influence ACPA-IgG_4_-Fc/ACPA-IgG_x_-Fc interactions, and that the distinct shift towards sialylated glycan species in the ACPA-IgG_4_ glycopeptide profile indicates a preference towards sialylated IgG_4_ glycans in such interactions. However, it is important to point out that similarly to ACPA, IgG rheumatoid factor (RF) has been reported to contain elevated levels of IgG_4_ [38], and that the “RF-mimicking activity” of IgG_4_ is a confounding factor when measuring IgG_4_-RF [37]. Likewise, the IgG_4_ in the ACPA fractions may not all necessarily be ACPAs, as antibodies with other specificities could have been co-purified due to their Fc-Fc-binding capacity [39].

The other main observation in the IgG_4_ glycan profile was the significantly lower abundance of FA2 in the ACPA eluate, compared to FT. Thus, an inverse trend compared to the IgG_1_ glycans is observed. However, this trend results in a more similar FA2 distribution in ACPA-IgG_4_ and ACPA-IgG_1_ (Table 1). At present we cannot be certain that this finding represents a specific ACPA glycan feature with specific FA2 functionality (independent of the IgG subclass), but it is an interesting possibility. Even though the distributions of FA2 are indeed similar among the ACPA isotypes, the overall profiles/substitution patterns (galactosylation, sialylation and bisected species), show major variations and are not shifting to a general homologous ACPA profile.

In addition to the significantly elevated FA2 levels in ACPA IgG_1_, two significant differences in glycan profiles were observed compared to FT IgG_1_: I) an under-representation of all bisected glycoforms, and II) a higher degree of all mono-antennary glycoforms (Figure 4, Table 1). Additionally, an under-representation of all afucosylated glycoforms was confirmed [10]. Noteworthy, in *in vitro* studies, both bisected- and core afucosylated Fc-glycans have been shown to increase the affinity to FcγII and III receptors, potentially resulting in more potent ADCC [14], [21], [22]. In accordance with our data, elevated levels of fucosylated N-glycans have previously been found in sera from RA patients, both for all serum proteins as well as specifically for IgG [24], [27]. Furthermore, a recent study demonstrated that ACPA-IgG_1_ Fc core-fucosylation was elevated compared to total IgG_1_ prior to RA disease onset, and then further elevated when the disease was initiated [10].

In contrast to the bisected and afucosylated glycans, little is known about the mono-antennary (A1) Fc glycans, and their effect on IgG functionality. Most likely this is due to the low amounts of these forms in IgG [20]. Furthermore, when analyzed by mass spectrometry, these glycans can be generated inside the instrument via in-source fragmentation. This could possibly explain the significantly increased amounts of FA1 in the ACPA fractions (since FA2 is also significantly increased), but does not explain the significantly higher abundance of FA1G1 (p = 0.04) and FA1G1S1 (p = 0.03) in ACPA-IgG_1_ compared to FT-IgG_1_. Further research is needed in order to investigate the potency and biological effect of these potentially important monoantennary glycans.

Due to the limited number (n = 4) of SF samples, no statistical comparisons were made between the SF- and S/P-samples in terms of Fc-glycan or IgG-isotype distribution. Individual values are provided in Table S3. Generally the shift from higher to lower (or lower to higher) distribution comparing FT and ACPA is the same independent of whether the ACPA was enriched from S/P or SF. However, from the acquired data we can conclude that high distributions of FA2 and mono-antennary forms, as well as low distributions of afucosylated and bisected forms, are most prominent in IgG_1_-SF and/or ACPA-IgG_1_-SF (Figure S6). Furthermore, it is likely that SF-IgG_4_ has a higher distribution of FA2 and a lower distribution of FA2G2S1 compared to S/P-IgG_4_ (Figure S6). Notable is also that the elevated levels of IgG_4_-ACPA is prominent in ACPA-S/P and that the IgG_2_ isotype distribution generally is low in both FT-SF and ACPA-SF (Figure S6). The relatively higher proportion of ACPA in the inflamed joints combined with the observation that as much as 30% of the total memory B-cell pool in RA SF is ACPA-specific, suggests a local autoantibody production in these compartments and an ongoing auto-immune response [30], [40], [41]. Hence, it may not be surprising that the glycoforms that are characteristic for ACPA-IgG_1_ (high: FA2 and monoantennary forms, as well as low: afucosylated and bisected forms) are particularly enhanced in the SF samples. On the contrary, the increased IgG_4_ levels in the ACPA eluted fractions which were particularly pronounced in S/P might indicate that IgG_4_ is having a different, peripheral and/or secondary function.

In addition to the variation in ACPA isotype and Fc-glycosylation pattern, a majority of the studied patients had significantly elevated levels (p = 0.0001) of one variable region λ-chain peptide in the ACPA (Figure 3). The old paradigm in immunological sciences states that antigen specificity is determined by a completely random process which will result in unique antigen binding peptide sequence regions in antibodies (of similar target) in different individuals. A number of studies, several of which were MS-based, have in recent years challenged this dogma [42]–[47]. Even though MS methods are not as sensitive as genomic studies, MS-based methodologies may still give a truer picture of the expression levels of the abundant IgG-peptide repertoire. It is possible that the increased abundance of this λ-peptide is yet another example indicating sequence homology between antibodies of particular specificity. It is important to point out that the identified peptide (DFMLTQPHSVSESPGK, Figure S3) is not part of any of the three complementary determining regions (CDRs). The sequence homology is from the first variable framework region of LV603 and LV601; the full sequences can be found at http://www.uniprot.org/ (UniProt IDs: P06317 and P01721). Noteworthy is that the λ-chains and DFMLTQPHSVSESPGK previously were found and identified in immunoglobulin λ light chain type amyloid fibril proteins [48], [49].

The sensitivity of our method further allowed us to detect and quantify in the ACPA and FT fractions traces of the following proteins (ACPA:FT): C1q (∼0.1%:0.03%), IgM (∼0.9%:0.1%), IgA (∼0.1%:0.03%) and CD5L (∼0.1%:0.02%), with numbers in parentheses referring to the relative abundance (total IgG = 100%). It is noteworthy that all four of these proteins were detected following both IgG and ACPA purifications, with significantly higher abundances in the ACPA fractions compared to FT (p<0.002, Table S4). The presence of these proteins can be explained by the likely formation of immune complexes. C1q as the initiator of the classical complement pathway [12], IgM and IgA as RF [50], and CD5L as associated with IgM [51], or potentially as a citrullinated ACPA target [52]. It should be pointed out that serum albumin was also detectable in the samples, but never exceeded 0.1% in abundance.

Compared to other studies investigating Fc-glycosylation patterns in ACPA, our methodology has the advantage of measuring IgG-isotype and peptide sequence distribution as well as IgG_x_-Fc glycan profiles simultaneously. As demonstrated herein, we succeeded in one single analysis in both confirming the results of multiple previous studies as well as obtaining novel data on the ACPA-IgG_4_-Fc glycosylation profile and new information on ACPA-IgG_1_ glycans. From the generated data, it is evident that IgG Fc-glycosylation patterns are highly complex but with particular distinct features. Hence, each glycan profile represents a specific pattern that likely affects different functions depending on the IgG-type and/or IgG-specificity. Increased knowledge of ACPA glycosylation profiles in different IgG isotypes will thus likely be important for future *in vitro* and *in vivo* functional studies. This knowledge should also be put in relation to the B-cell status (activation, differentiation, cytokine and T-cell effects, localization, etc.). Combined, such knowledge can improve our understanding of disease mechanisms and pathways in ACPA-positive RA. Prospectively, the IgG Fc-glycosylation profile could potentially be used for clinical diagnostics and prognostic purposes.

## Supporting Information

Figure S1 **Extracted ion chromatograms.** (**A**) Extracted ion chromatograms of FA2, FA2G1 and FA2G2 glycopeptides, from ACPA and FT IgG_1_, IgG_2/3_ and IgG_4/(3)_ from subject 1. The solid lines indicate integrated ions from glycopeptides with one misscleavage. (**B**) Extracted ion chromatograms of merged FA2, FA2G1 and FA2G2 glycopeptide ions of IgG_2_ (EEQ**F**NST**F**R), IgG_3_ (EEQ**F**NST**F**R or EEQ**Y**NST**F**R) and IgG_4_, (EEQ**F**NST**Y**R) from the ACPA and the FT extracted S/P pool following protein A column separation of IgG_3_ (found in the protein A FT fraction). It was concluded that the majority of IgG_3_ has the EEQ**F**NST**F**R sequence since EEQ**F**NST**F**R and not EEQ**Y**NST**F**R was the main glycopeptide found in the protein A FT.
(TIF)

Figure S2 **Multivariate analysis scores plots of the SF samples based on both the glycan and protein data.** Subjects are labeled according to Table S1. (**A**) PCA model constructed from two components (R^2^ = 0.34, Q^2^ = 0.03). Samples cluster according to individual. (**B**) OPLS-DA model constructed from two components (R^2^ = 0.97, Q^2^ = 0.70). Samples separate distinctly along the x-axis according to FT and ACPA specificity.
(TIF)

Figure S3 **MS/MS peptide spectra.** Spectra were obtained from precursors m/z 880.4204 (SF-ACPA) and m/z 880.4191 (S/P-ACPA) corresponding to [M+2]^2+^ of DFMLTQPHSVSEPGK. Assigned b- and y-ions are indicated in the figure.
(TIF)

Figure S4 **IgG_1_-Fc-glycan distribution and intra-individual differences in bisected (n = 5), afucosylated (n = 4) and mono-antennary (n = 3) forms.** Shown p-values were obtained using paired Student’s T-test. S/P samples (gray), SF samples (red).
(TIF)

Figure S5 **Fc-glycan distribution and intra-individual differences of FA2 in IgG_1_ and IgG_4/(3)_, as well as FA2G2S1 in IgG_4/(3)_.** Shown p-values were obtained using paired Student’s T-test. S/P samples (gray), SF samples (red).
(TIF)

Figure S6 **Mean and standard error of mean (SEM) of the different sample types.** Data include control IgG standard (Sigma Aldrich) run in duplicates in 0.3 to 2 pmol/5 uL injections (n = 10) as well as FT-S/P, ACPA-S/P, FT-SF and ACPA-SF samples, respectively. (**A**) FA2 distribution in IgG_1_. (**B**) Sum of mono-antennary form in IgG_1_. (**C**) Sum of afucosylated forms in IgG_1_. (**D**) Sum of bisected forms in IgG_1_. (**E**) FA2 distribution in IgG_4_. (**F**) FA2G2S1 distribution in IgG_4_. (**G**) IgG_4_ isotype distribution. (**H**) IgG_2_ isotype distribution.
(TIF)

Table S1 **Clinical data of participating subjects.** Disease duration times and symptoms duration times were based on criteria’s given by Raza et al [29] according to “Initial fulfillment of RA criteria based on rheumatologist's assessment” and “First musculoskeletal symptoms relevant, (in the opinion of the assessing rheumatologist), to the current complaint”, respectively. Subject 15–17 were sampled at two occasions, approximately 1 year between sampling dates. In order to investigate potential differences between the blood matrixes, both plasma and serum extracted ACPA and FT were obtained from subject 9. The correlation between the measured glycan and protein levels in the serum and plasma FT was very good (R^2^ = 0.99). Two outliers in the ACPA samples (HV301 and HV308) affected the overall correlation, (R^2^ = 0.66 compared to R^2^ = 0.94, if the outliers were excluded).
(DOCX)

Table S2 **Glycopeptide ions searched for.** In total 19 glycan structures substituting 6 different peptides (determined by monoisotopic mass and retention times) were searched for via two charge states.
(DOCX)

Table S3 **Individual ACPA and FT Fc-glycan distributions (%) and protein abundances.** Protein abundances are given as log_10_[ion intensity/average ion intensity]. Values from IgG control standard (Sigma Aldrich) are also included. D: detected, -: not detected.
(XLSX)

Table S4 **List of quantified IgG chains/peptides and other proteins found in the proteomics analysis of the FT and ACPA samples.** Protein levels, (normalized to the average abundance [31] and log-transformed), and their respective standard deviations are shown. P-values comparing FT and ACPA were obtained with paired t-test (p<5.0E-2 is significant, bolded). Peptide sequences are given in Table S5. LV603, CD5L, KV106, LAC3, HV320, HV308 were not found in subject 17 and 18. LV102 was not found in any of the SF samples (subject 15–18).
(DOCX)

Table S5 **Identified peptide sequences.**
(DOCX)
