# Supplementary figures and images for: IgG Antibodies to Cyclic Citrullinated Peptides Exhibit Profiles Specific in Terms of IgG Subclasses, Fc-Glycans and a Fab-Peptide Sequence
Source: PLoS One. 2014 Nov 26;9(11):e113924. doi: 10.1371/journal.pone.0113924 (PMC4245247; doi:10.1371/journal.pone.0113924)

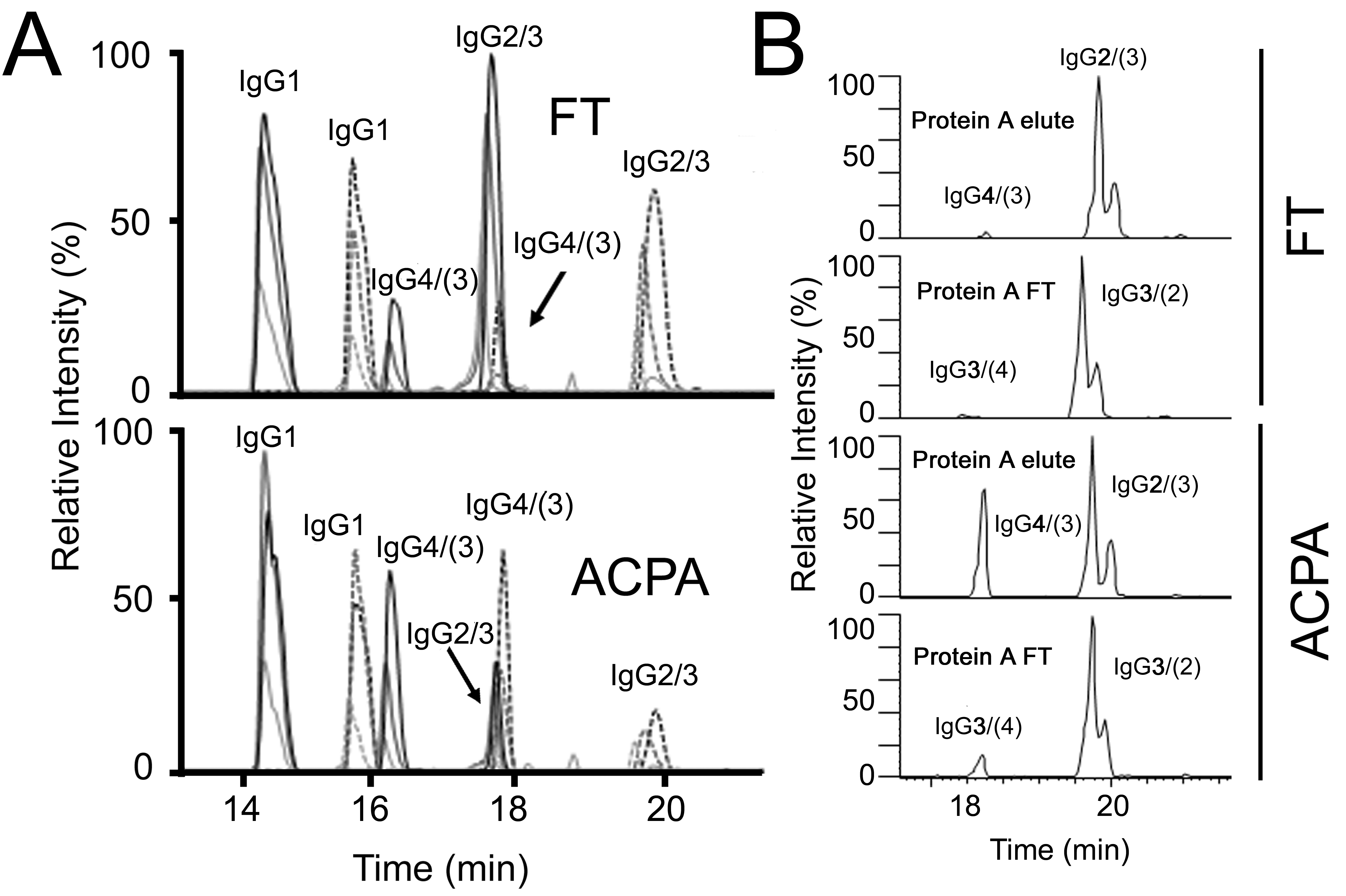

Supplement: Figure S1 — Extracted ion chromatograms. (A) Extracted ion chromatograms of FA2, FA2G1 and FA2G2 glycopeptides, from ACPA and FT IgG1, IgG2/3 and IgG4/(3) from subject 1. The solid lines indicate integrated ions from glycopeptides with one misscleavage. (B) Extracted ion chromatograms of merged FA2, FA2G1 and FA2G2 glycopeptide ions of IgG2 (EEQFNSTFR), IgG3 (EEQFNSTFR or EEQYNSTFR) and IgG4, (EEQFNSTYR) from the ACPA and the FT extracted S/P pool following protein A column separation of IgG3 (found in the protein A FT fraction). It was concluded that the majority of IgG3 has the EEQFNSTFR sequence since EEQFNSTFR and not EEQYNSTFR was the main glycopeptide found in the protein A FT. (TIF) [file pone.0113924.s001.tif]

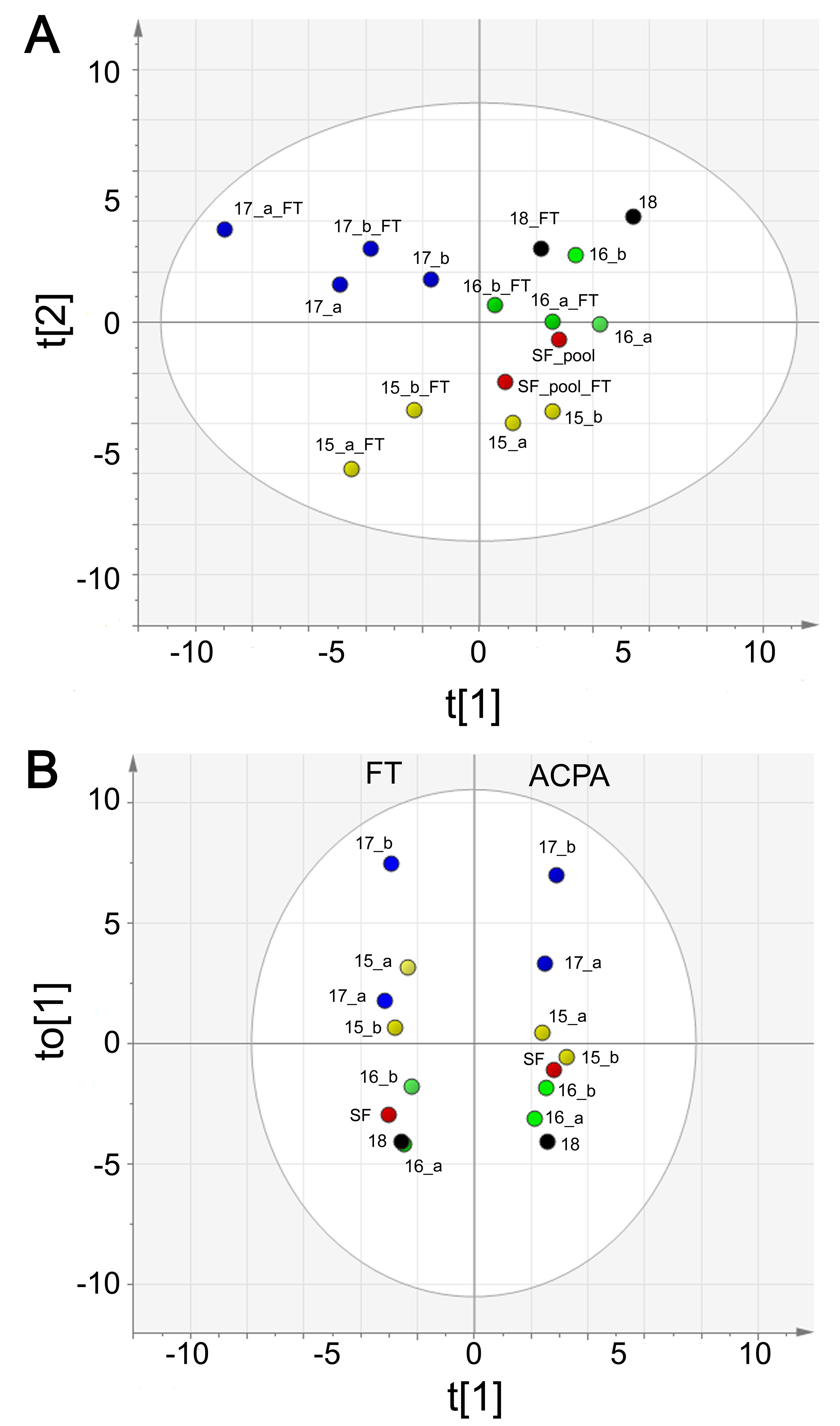

Supplement: Figure S2 — Multivariate analysis scores plots of the SF samples based on both the glycan and protein data. Subjects are labeled according to Table S1. (A) PCA model constructed from two components (R2 = 0.34, Q2 = 0.03). Samples cluster according to individual. (B) OPLS-DA model constructed from two components (R2 = 0.97, Q2 = 0.70). Samples separate distinctly along the x-axis according to FT and ACPA specificity. (TIF) [file pone.0113924.s002.tif]

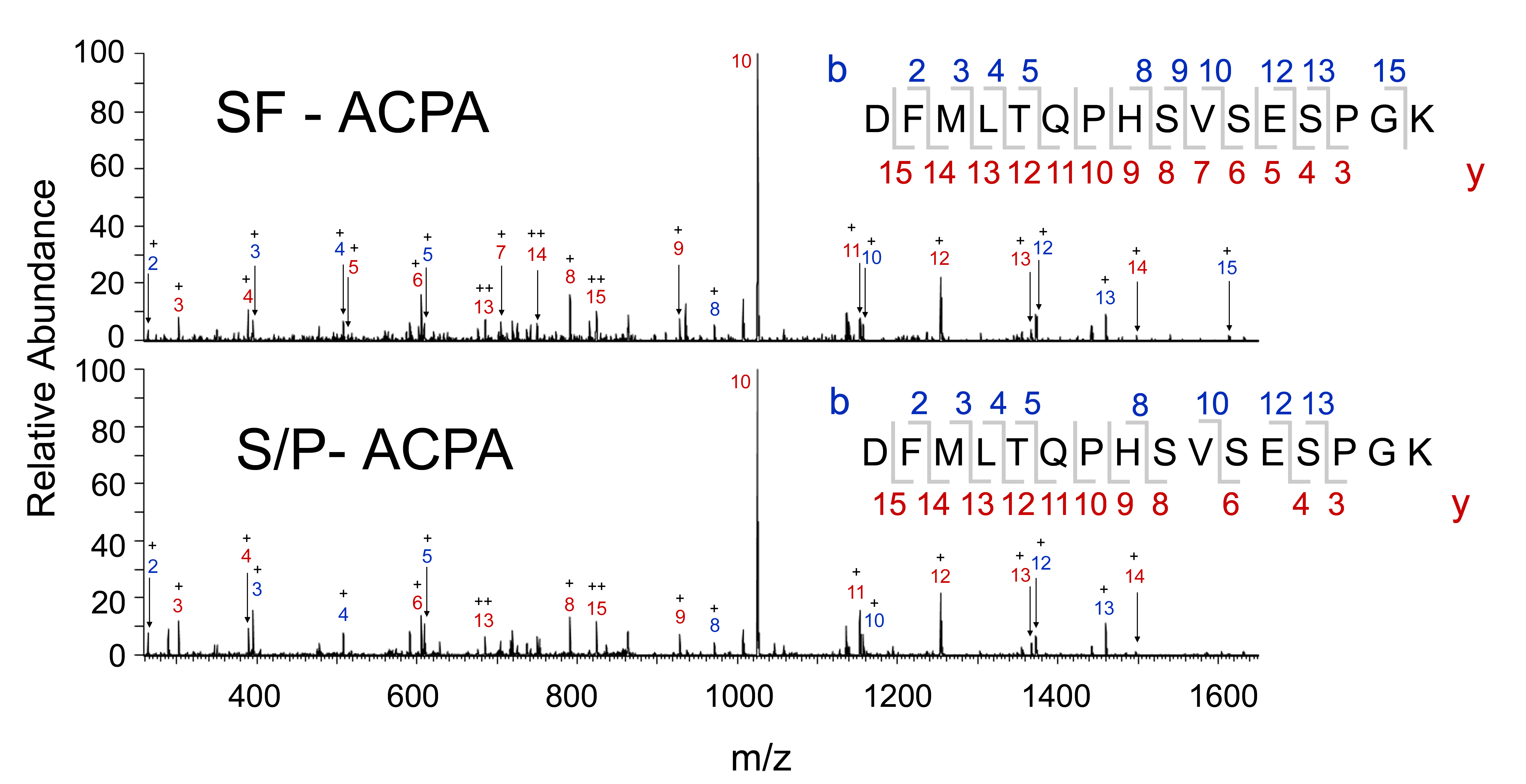

Supplement: Figure S3 — MS/MS peptide spectra. Spectra were obtained from precursors m/z 880.4204 (SF-ACPA) and m/z 880.4191 (S/P-ACPA) corresponding to [M+2]2+ of DFMLTQPHSVSEPGK. Assigned b- and y-ions are indicated in the figure. (TIF) [file pone.0113924.s003.tif]

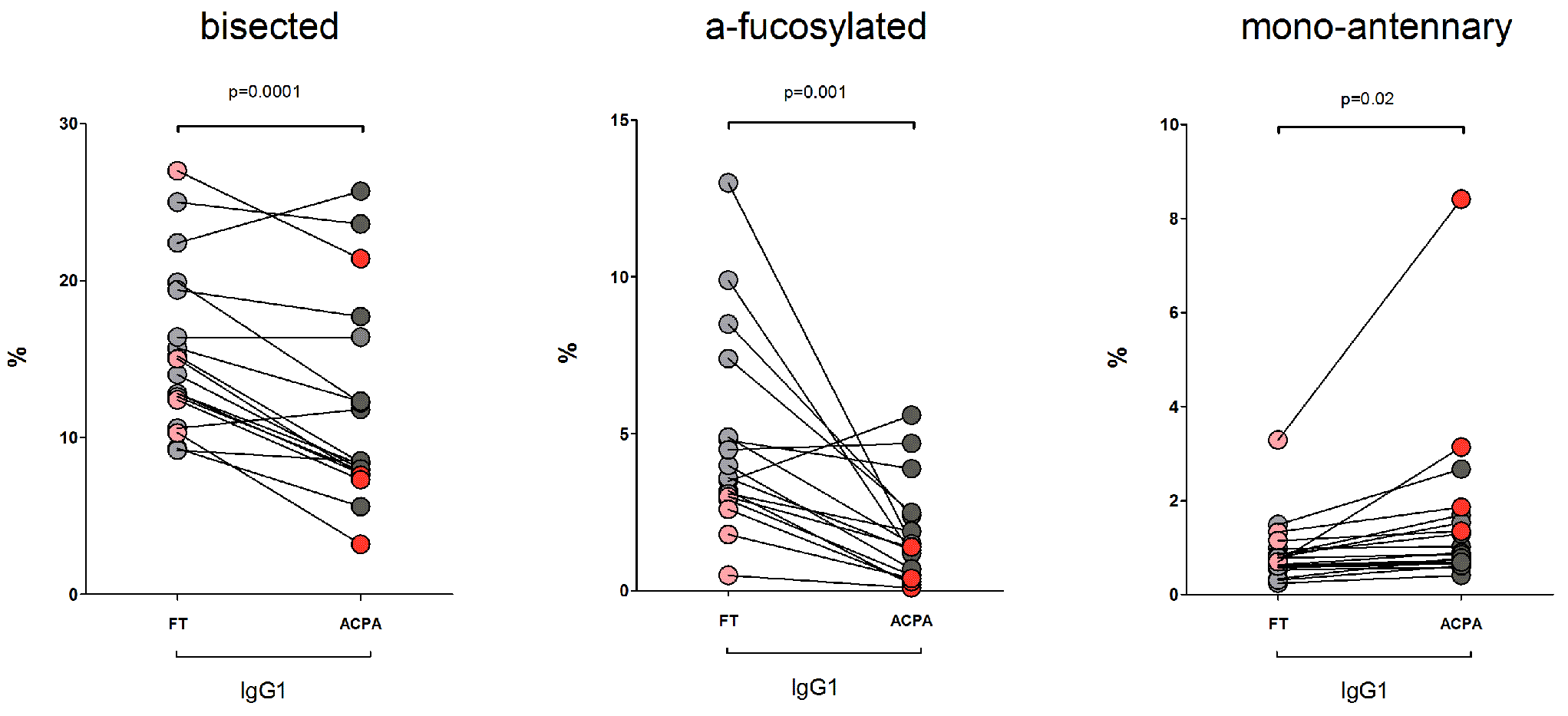

Supplement: Figure S4 — IgG1-Fc-glycan distribution and intra-individual differences in bisected (n = 5), afucosylated (n = 4) and mono-antennary (n = 3) forms. Shown p-values were obtained using paired Student’s T-test. S/P samples (gray), SF samples (red). (TIF) [file pone.0113924.s004.tif]

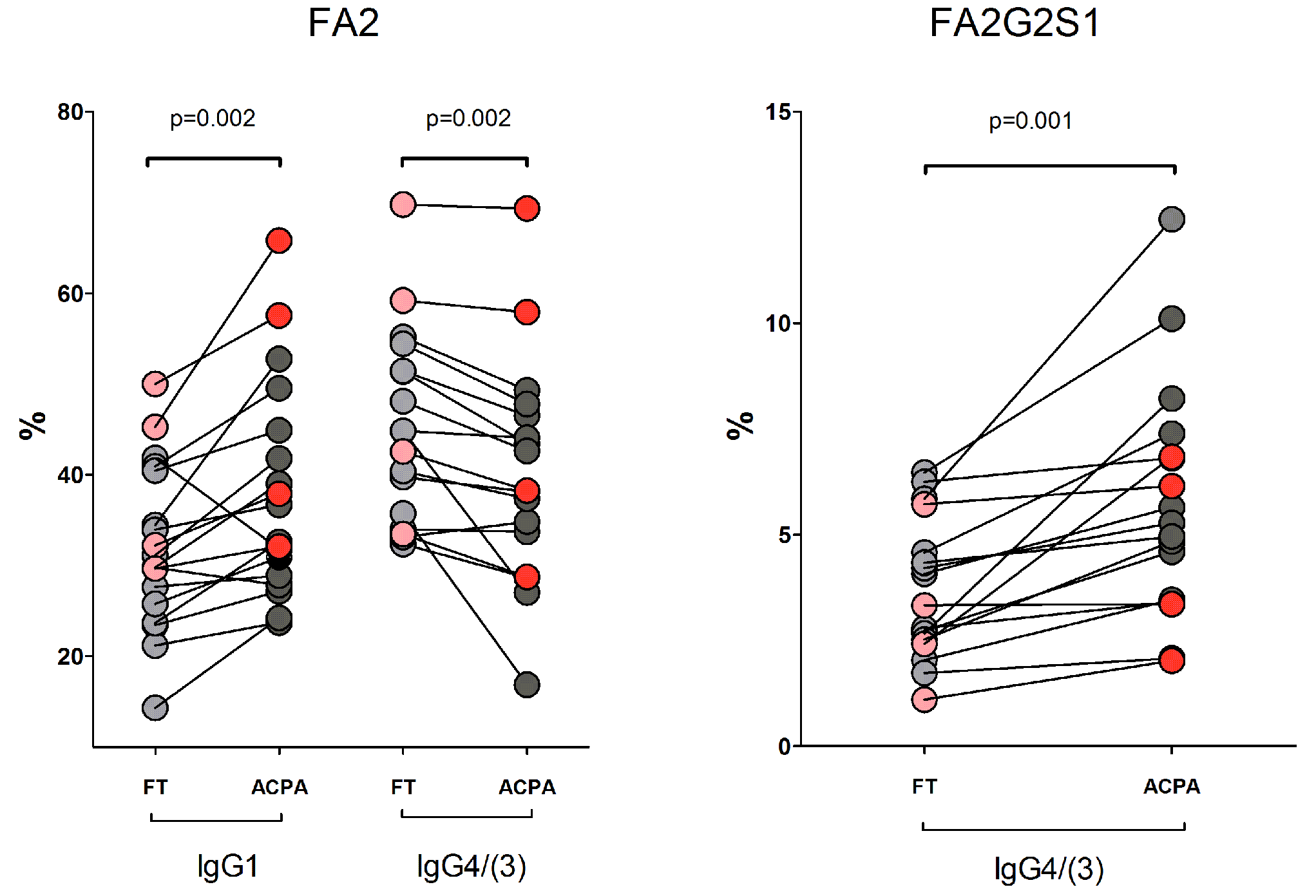

Supplement: Figure S5 — Fc-glycan distribution and intra-individual differences of FA2 in IgG1 and IgG4/(3), as well as FA2G2S1 in IgG4/(3). Shown p-values were obtained using paired Student’s T-test. S/P samples (gray), SF samples (red). (TIF) [file pone.0113924.s005.tif]

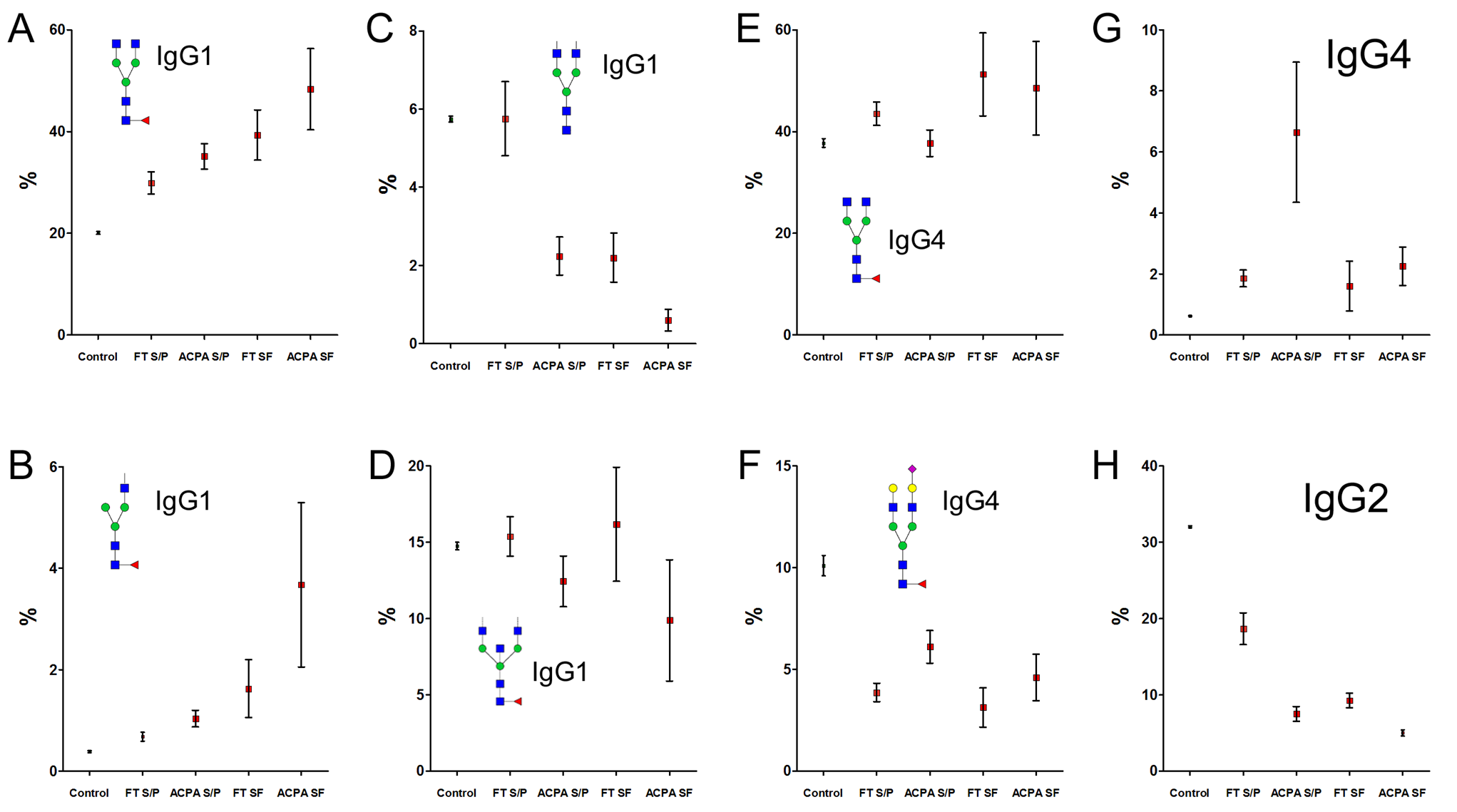

Supplement: Figure S6 — Mean and standard error of mean (SEM) of the different sample types. Data include control IgG standard (Sigma Aldrich) run in duplicates in 0.3 to 2 pmol/5 uL injections (n = 10) as well as FT-S/P, ACPA-S/P, FT-SF and ACPA-SF samples, respectively. (A) FA2 distribution in IgG1. (B) Sum of mono-antennary form in IgG1. (C) Sum of afucosylated forms in IgG1. (D) Sum of bisected forms in IgG1. (E) FA2 distribution in IgG4. (F) FA2G2S1 distribution in IgG4. (G) IgG4 isotype distribution. (H) IgG2 isotype distribution. (TIF) [file pone.0113924.s006.tif]
