# Supplementary material for: IgG Antibodies to Cyclic Citrullinated Peptides Exhibit Profiles Specific in Terms of IgG Subclasses, Fc-Glycans and a Fab-Peptide Sequence
Source: PLoS One. 2014 Nov 26;9(11):e113924. doi: 10.1371/journal.pone.0113924 (PMC4245247; doi:10.1371/journal.pone.0113924)
Supplement: Table S1 — Clinical data of participating subjects. Disease duration times and symptoms duration times were based on criteria’s given by Raza et al [29] according to “Initial fulfillment of RA criteria based on rheumatologist's assessment” and “First musculoskeletal symptoms relevant, (in the opinion of the assessing rheumatologist), to the current complaint”, respectively. Subject 15–17 were sampled at two occasions, approximately 1 year between sampling dates. In order to investigate potential differences between the blood matrixes, both plasma and serum extracted ACPA and FT were obtained from subject 9. The correlation between the measured glycan and protein levels in the serum and plasma FT was very good (R2 = 0.99). Two outliers in the ACPA samples (HV301 and HV308) affected the overall correlation, (R2 = 0.66 compared to R2 = 0.94, if the outliers were excluded). (DOCX) [file pone.0113924.s007.docx]

**Table S1.** Clinical data of participating subjects. Disease duration times and symptoms duration times were based on criteria’s given by Raza et al ([1](#_ENREF_1)) according to "Initial fulfillment of RA criteria based on rheumatologist´s assessment" and “First musculoskeletal symptoms relevant, (in the opinion of the assessing rheumatologist), to the current complaint”, respectively. Subject 15-17 were sampled at two occasions, approximately 1 year between sampling dates. In order to investigate potential differences between the blood matrixes, both plasma and serum extracted ACPA and FT were obtained from subject 9. The correlation between the measured glycan and protein levels in the serum and plasma FT was very good (R^2^=0.99). Two outliers in the ACPA samples (HV301 and HV308) affected the overall correlation, (R^2^=0.66 compared to R^2^=0.94, if the outliers were excluded).

| Subject | Gender^a^ | Age^b^ | Sample type | Disease duration^c^ | Symptom duration^d^ |
| --- | --- | --- | --- | --- | --- |
| 1 | M | 77 | Serum | 28 | 30 |
| 2 | F | 53 | Serum | 25 | 28 |
| 3 | F | 63 | Serum | 1 | 1 |
| 4 | M | 67 | Serum | 1 | 2 |
| 5 | F | 68 | Serum | 44 | 47 |
| 6 | F | 61 | Serum | 2 | 2 |
| 7 | M | 29 | Plasma | 2 | 2 |
| 8 | M | 53 | Serum | 2 | 2 |
| 9 | M | 55 | Serum/Plasma^e^ | 40 | 41 |
| 10 | F | 41 | Plasma | 1 | 4 |
| 11 | M | 73 | Plasma | 21 | 24 |
| 12 | F | 22 | Plasma | 0 | 3 |
| 13 | M | 58 | Plasma | 0 | 1 |
| 14 | M | 65 | Plasma | 24 | 24 |
| 15_a | F | 42 | Synovial Fluid | 19 | 22 |
| 15_b | F | 43 | Synovial Fluid | 20 | 23 |
| 16_a | M | 52 | Synovial Fluid | 0 | 0.5 |
| 16_b | M | 53 | Synovial Fluid | 1 | 1.5 |
| 17_a | F | 47 | Synovial Fluid | 16 | 17 |
| 17_b | F | 48 | Synovial Fluid | 17 | 18 |
| 18 | M | 69 | Synovial Fluid | 15 | 16 |

^a^Female (F), Male (M); ^b-d^Years; ^e^Both serum and plasma was collected from this individual 1. Raza K, Saber TP, Kvien TK, Tak PP, Gerlag DM. Timing the therapeutic window of opportunity in early rheumatoid arthritis: proposal for definitions of disease duration in clinical trials. Ann Rheum Dis. 2012;71:1921-3.
