# Supplementary material for: IgG Antibodies to Cyclic Citrullinated Peptides Exhibit Profiles Specific in Terms of IgG Subclasses, Fc-Glycans and a Fab-Peptide Sequence
Source: PLoS One. 2014 Nov 26;9(11):e113924. doi: 10.1371/journal.pone.0113924 (PMC4245247; doi:10.1371/journal.pone.0113924)
Supplement: Table S2 — Glycopeptide ions searched for. In total 19 glycan structures substituting 6 different peptides (determined by monoisotopic mass and retention times) were searched for via two charge states. (DOCX) [file pone.0113924.s008.docx]

**Table S2.** Glycopeptides searched for via Quanti ([1](#_ENREF_1)). In total 19 glycan structures substituting 6 different peptides (determined by monoisotopic mass and retention times) were searched for via two charge states.

| Peptides: IgG type; charges | Glycoforms |
| --- | --- |
| EEQ**Y**NST**Y**R: IgG_1_; 2+ and 3+ | A2 |
| EEQ**F**NST**F**R: IgG_2/3_; 2+ and 3+ | A2G1 |
| EEQ**F**NST**Y**R /(EEQ**Y**NST**F**R): IgG_4/(3)_; 2+ and 3+ | A2G2 |
|  | A2B |
| TKPREEQ**Y**NST**Y**R: IgG_1_; 3+ and 4+ | FA1 |
| TKPREEQ**F**NST**F**R: IgG_2/3_; 3+ and 4+ | FA1G1 |
| TKPREEQ**F**NST**Y**R /(TKPREEQ**Y**NST**F**R): IgG_4/(3)_; 3+ and 4+ | FA1G1S1 |
|  | FA2 |
|  | FA2G1 |
|  | FA2G2 |
|  | FA2G1S1 |
|  | FA2G2S1 |
|  | FA2G2S2 |
|  | FA2B |
|  | FA2BG1 |
|  | FA2BG2 |
|  | FA2BG1S1 |
|  | FA2BG2S1 |
|  | FA2BG2S2 |

1. Lundstrom SL, Yang H, Lyutvinskiy Y, Rutishauser D, Herukka SK, Soininen H, et al. Blood Plasma IgG Fc Glycans are Significantly Altered in Alzheimer's Disease and Progressive Mild Cognitive Impairment. J Alzheimers Dis. 2014;38:567-79.
