# Supplementary material for: IgG Antibodies to Cyclic Citrullinated Peptides Exhibit Profiles Specific in Terms of IgG Subclasses, Fc-Glycans and a Fab-Peptide Sequence
Source: PLoS One. 2014 Nov 26;9(11):e113924. doi: 10.1371/journal.pone.0113924 (PMC4245247; doi:10.1371/journal.pone.0113924)
Supplement: Table S4 — List of quantified IgG chains/peptides and other proteins found in the proteomics analysis of the FT and ACPA samples. Protein levels, (normalized to the average abundance [31] and log-transformed), and their respective standard deviations are shown. P-values comparing FT and ACPA were obtained with paired t-test (p<5.0E-2 is significant, bolded). Peptide sequences are given in Table S5. LV603, CD5L, KV106, LAC3, HV320, HV308 were not found in subject 17 and 18. LV102 was not found in any of the SF samples (subject 15–18). (DOCX) [file pone.0113924.s010.docx]

**Table S4.** List of quantified IgG chains/peptides and other proteins found in the proteomics analysis of the FT and ACPA samples. Protein levels, (normalized to the average abundance ([1](#_ENREF_1)) and log-transformed), and their respective standard deviations are shown. P-values comparing FT and ACPA were obtained with paired t-test (p<5.0E-2 is significant, bolded). Peptide sequences are given in Table S5. LV603, CD5L, KV106, LAC3, HV320, HV308 were not found in subject 17 and 18. LV102 were not found in any of the SF samples (subject 15-18).

| Abbreviation | Full name | UniProt ID | S/P |  | SF |  | p-value (paired) | | | | |
| --- | --- | --- | --- | --- | --- | --- | --- | --- | --- | --- | --- |
|  |  |  | FT | ACPA | FT | ACPA | S/P (n=14) | S/P+SF (n=18) | | | |
|  |  |  | AV^a^±STD^b^ | AV±STD | AV±STD | AV±STD | FT/ACPA | FT/ACPA | | | |
|  |  |  |  |  |  |  | Significant increase | | | | |
| C1Q^c^ | Complement C1q | P02745/6/7^c^ | 0.3±0.2 | 1±0.2 | -0.2±0.1 | 0.3±0.6 | **4.9E-06** | | **3.7E-06** | | |
| IGHM | Ig mu chain C region | P01871 | -0.4±0.3 | 0.6±0.4 | -0.1±0.3 | 0.2±0.5 | **7.2E-07** | | **7.3E-06** | | |
| IGHA1 | Ig α-1 chain C region | P01876 | -0.4±0.3 | 0.2±0.3 | 0.1±0.3 | 0.3±0.4 | **4.5E-06** | | **6.6E-05** | | |
| LV603/ LV601^d^ | Ig l-chain V-VI region SUT/ AR | P06317/1721^d^ | -0.5±0.3 | 0.04±0.4 | -0.03±0.2 | 1±0.5 | **5.8E-04** | | **1.4E-04** | | |
| IGHG4 | Ig g-4 chain C region | P01861 | -0.1±0.2 | 0.3±0.5 | -0.1±0.3 | 0.2±0.3 | **3.2E-03** | | **6.0E-04** | | |
| CD5L | CD5 antigen-like | O43866 | -0.01±0.3 | 0.4±0.4 | 0.2±0.1 | 0.6±0.1 | **4.6E-03** | | **1.6E-03** | | |
| IGHG1 | Ig g-1 chain C region | P01857 | -0.05±0.1 | 0.1±0.1 | -0.01±0.02 | 0.04±0.1 | **9.2E-03** | | **4.7E-03** | | |
| IGHG3 | Ig g-3 chain C region | P01860 | -0.1±0.2 | 0.1±0.3 | -0.01±0.1 | 0.1±0.2 | **2.7E-02** | | **1.9E-02** | | |
|  |  |  |  |  |  |  | Significant decrease | | | | |
| IGHG2 | Ig g-2 chain C region | P01859 | 0.2±0.1 | -0.1±0.2 | 0.1±0.1 | -0.1±0.1 | **2.4E-05** | | **3.0E-06** | |  |
| LV302 | Ig l-chain V-III region LOI | P80748 | 0.2±0.1 | -0.2±0.4 | 0.3±0.1 | 0.1±0.3 | **2.4E-04** | | **6.8E-05** | |  |
| KV119 | Ig k-chain V-I region Wes | P01611 | 0.3±0.3 | -0.2±0.6 | 0.3±0.2 | -0.4±0.5 | **7.9E-03** | | **8.6E-04** | |  |
| HV304 | Ig heavy chain V-III region TIL | P01765 | 0.1±0.2 | -0.1±0.2 | 0.3±0.1 | -0.1±0.4 | **2.9E-03** | | **1.4E-03** | |  |
| KV106 | Ig k-chain V-I region EU | P01598 | 0.1±0.2 | -0.1±0.4 | 1±1 | -0.1±0.2 | 2.1E-01 | | **1.9E-02** | |  |
| LV301 | Ig l chain V-III region SH | P01714 | 0.04±0.4 | -0.2±0.3 | 0.5±0.3 | -0.1±0.5 | 1.2E-01 | | **2.2E-02** | |  |
| KV204 | Ig k-chain V-II region TEW | P01617 | -0.001±0.2 | -0.3±0.6 | 0.2±0.3 | 0.02±0.3 | **2.8E-02** | | **2.8E-02** | |  |
|  |  |  |  |  |  |  | Not significant | | |  |  |
| LAC1/ IGLL5^e^ | Ig l-1 chain C regions/ Ig-l-like polypeptide 5 | P0CG04/ B9A064^e^ | 0.03±0.1 | 0.1±0.2 | -0.1±0.05 | 0.02±0.3 | 1.2E-01 | | 1.2E-01 | |  |
| LAC3 | Ig l-3 chain C regions | P0CG06 | -0.04±0.2 | -0.01±0.3 | -0.1±0.1 | 0.2±0.1 | 4.5E-01 | | 1.2E-01 | |  |
| LV403 | Ig l-chain V-IV region Hil | P01717 | -0.004±0.1 | -0.01±0.6 | 0.3±0.2 | -0.2±0.2 | 1.0E+00 | | 4.6E-01 | |  |
| HV305 | Ig heavy chain V-III region BRO | P01766 | -0.04±0.2 | 0.1±0.6 | 0.1±0.3 | 0.04±0.2 | 4.8E-01 | | 6.3E-01 | |  |
| KV101 | Ig k-chain V-I region AG | P01593 | -0.002±0.2 | 0.1±0.5 | 0.3±0.3 | -0.1±0.2 | 7.1E-01 | | 6.6E-01 | |  |
| HV320 | Ig heavy chain V-III region GAL | P01781 | 0.1±0.3 | 0.2±0.4 | 0.1±0.2 | -0.1±0.04 | 6.8E-01 | | 8.9E-01 | |  |
| HV209/HV207^f^ | Ig heavy chain V-II region ARH-77/ NEWM | P06331/1825^f^ | 0.1±0.3 | 0.1±0.4 | 0.4±0.5 | -0.1±0.3 | 6.5E-01 | | 6.3E-01 | |  |
| HV308 | Ig heavy chain V-III region GA | P01769 | 0.1±0.4 | 0.1±0.5 | -1±1 | -0.03±0.5 | 9.4E-01 | | 6.2E-01 | |  |
| KV402 | Ig k chain V-IV region Len | P01625 | -0.1±0.1 | -0.02±0.5 | 0.1±0.1 | 0.2±0.2 | 8.4E-01 | | 6.1E-01 | |  |
| LV102 | Ig l-chain V-I region HA | P01700 | 0.1±0.3 | 0.01±0.4 | - | - | 5.9E-01 | | 5.9E-01 | |  |
| KV305 | Ig k-chain V-III region WOL | P01623 | -0.02±0.3 | -0.04±0.4 | 0.2±0.1 | -0.1±0.1 | 8.3E-01 | | 3.0E-01 | |  |
| HV301 | Ig heavy chain V-III region TRO | P01762 | 0.2±0.2 | 0.01±0.5 | 0.1±0.2 | 0.1±0.2 | 2.0E-01 | | 3.0E-01 | |  |
| IGKC | Ig k-chain C region | P01834 | 0.04±0.1 | -0.04±0.1 | 0.02±0.04 | 0.03±0.04 | 5.8E-02 | | 7.4E-02 | |  |
| HV107/ HV106^g^ | Ig heavy chain V-I region Mot/ SIE | P06326/1761^g^ | 0.2±0.2 | 0.1±0.6 | 1±1 | 0.3±1 | 3.6E-01 | | 5.2E-02 | |  |

Abbreviations: ^a^Average; ^b^Standard Deviation, ^c^Sum of C1q subcomponents A (P02745), B (P02746) and C (P02747), ^d^LV603 (P06317) and LV601 (P01721), ^e,f,g^LAC1 (P0CG04) and IGLL5 (B9A064), HV107 (P06326) and HV106 (P01761) and HV209 (P06331) and HV207 (P01825), could not be distinguished due to sequence homology

**References**

1. Lyutvinskiy Y, Yang H, Rutishauser D, Zubarev RA. In silico instrumental response correction improves precision of label-free proteomics and accuracy of proteomics-based predictive models. Mol Cell Proteomics. 2013;12:2324-31.
