# Supplementary material for: IgG Antibodies to Cyclic Citrullinated Peptides Exhibit Profiles Specific in Terms of IgG Subclasses, Fc-Glycans and a Fab-Peptide Sequence
Source: PLoS One. 2014 Nov 26;9(11):e113924. doi: 10.1371/journal.pone.0113924 (PMC4245247; doi:10.1371/journal.pone.0113924)
Supplement: Table S5 — Identified peptide sequences. (DOCX) [file pone.0113924.s011.docx]

**Table S5.** Identified peptide sequences.

| **Abbreviation** | | | | **UniProt ID** | | | **Identified Sequence** | | | | | | | **Homology** | | | **Mascot Scores** |  |  |
| --- | --- | --- | --- | --- | --- | --- | --- | --- | --- | --- | --- | --- | --- | --- | --- | --- | --- | --- | --- |
| IGHM | | | | P01871 | |  | EGKQVGSGVTTDQVQAEAK | | | | | | |  | | | 119 |  |  |
|  | | | |  | |  | QVGSGVTTDQVQAEAK | | | | | | |  | | | 115 |  |  |
|  | | | |  | |  | NVPLPVIAELPPK | | | | | | |  | | | 59 |  |  |
|  | | | |  | |  | YAATSQVLLPSK | | | | | | |  | | | 87 |  |  |
|  | | | |  | |  | YAATSQVLLPSKDVMQGTDEHVVCK | | | | | | |  | | | 85 |  |  |
|  | | | |  | |  | GGKYAATSQVLLPSKDVMQGTDEHVVCK | | | | | | |  | | | 68 |  |  |
|  | | | |  | |  | DVMQGTDEHVVCK | | | | | | |  | | | 82 |  |  |
| C1Q | C1QA | | | P02745 | | | KGHIYQGSEADSVFSGFLIFPSA | | | | | | |  | | | 83 |  |  |
|  |  | | |  | | | GHIYQGSEADSVFSGFLIFPSA | | | | | | |  | | | 86 |  |  |
|  |  | | |  | | | GLFQVVSGGMVLQLQQGDQVWVEKDPK | | | | | | |  | | | 84 |  |  |
|  | C1QB | | | P02746 | | | FDHVITNMNNNYEPR | | | | | | |  | | | 69 |  |  |
|  |  | | |  | | | IAFSATR | | | | | | |  | | | 63 |  |  |
|  |  | | |  | | | LEQGENVFLQATDK | | | | | | |  | | | 88 |  |  |
|  | C1QC | | | P027467 | | | FNAVLTNPQGDYDTSTGK | | | | | | |  | | | 93 |  |  |
|  |  | | |  | | | FQSVFTVTR | | | | | | |  | | | 61 |  |  |
|  |  | | |  | | | QKFQSVFTVTR | | | | | | |  | | | 51 |  |  |
|  |  | | |  | | | TNQVNSGGVLLR | | | | | | |  | | | 77 |  |  |
| IGHA1 | | | | P01876 | |  | DASGVTFTWTPSSGK | | | | | | |  | | | 82 |  |  |
|  | |  | | | |  | DLCGCYSVSSVLPGCAEPWNHGK | | | | | | |  | | | 59 |  |  |
|  | |  | | | |  | KGDTFSCMVGHEALPLAFTQK | | | | | | | IGHA2 (P01877) | | | 60 |  |  |
|  | |  | | | |  | QEPSQGTTTFAVTSILR | | | | | | | IGHA2 (P01877) | | | 118 |  |  |
|  | |  | | | |  | SGNTFRPEVHLLPPPSEELALNELVTLTCLAR | | | | | | | IGHA2 (P01877) | | | 99 |  |  |
|  | |  | | | |  | TFTCTAAYPESK | | | | | | |  | | | 71 |  |  |
|  | |  | | | |  | VAAEDWKKGDTFSCMVGHEALPLAFTQK | | | | | | | IGHA2 (P01877) | | | 87 |  |  |
|  | |  | | | |  | WLQGSQELPR | | | | | | | IGHA2 (P01877) | | | 64 |  |  |
| LV603 / LV601 | | | | P06317/1721 | | | DFMLTQPHSVSESPGK | | | | | | |  | | | 72 |  |  |
| IGHG4 | | | | P01861 |  | | DYFPEPVTVSWNSGALTSGVHTFPAVLQSSGLYSLSSVVTVPSSSLGTK | | | | | | | | 59 | | | | |
|  | | |  | | |  | EPQVYTLPPSQEEMTK | | | | | | |  | | | 61 |  |  |
|  | | |  | | |  | GFYPSDIAVEWESNGQPENNYKTTPPVLDSDGSFFLYSR | | | | | | |  | | | 91 |  |  |
|  | | |  | | |  | TPEVTCVVVDVSQEDPEVQFNWYVDGVEVHNAK | | | | | | |  | | | 119 |  |  |
|  | | |  | | |  | TTPPVLDSDGSFFLYSR | | | | | | |  | | | 83 |  |  |
|  | | |  | | |  | TYTCNVDHKPSNTK | | | | | | |  | | | 75 |  |  |
|  | | |  | | |  | TYTCNVDHKPSNTKVDK | | | | | | |  | | | 72 |  |  |
|  | | |  | | |  | TYTCNVDHKPSNTKVDKR | | | | | | |  | | | 64 |  |  |
|  | | |  | | |  | VSNKGLPSSIEK | | | | | | |  | | | 56 |  |  |
|  | | |  | | |  | YGPPCPSCPAPEFLGGPSVFLFPPKPK | | | | | | |  | | | 79 |  |  |
| CD5L | | | O43866 | | |  | CSGEEQSLEQCQHR | | | | | | |  | | | 64 |  |  |
|  | | |  | | |  | EATLQDCPSGPWGK | | | | | | |  | | | 52 |  |  |
|  | | |  | | |  | ELGCGAASGTPSGILYEPPAEK | | | | | | |  | | | 78 |  |  |
|  | | |  | | |  | GQWGTVCDDGWDIK | | | | | | |  | | | 57 |  |  |
|  | | |  | | |  | HQNQWYTVCQTGWSLR | | | | | | |  | | | 57 |  |  |
|  | | |  | | |  | IWLDNVR | | | | | | |  | | | 50 |  |  |
|  | | |  | | |  | LVGGDNLCSGR | | | | | | |  | | | 76 |  |  |
| IGHG1 | | | P01857 | | |  | EPQVYTLPPSRDELTK | | | | | | |  | | | 82 |  |  |
|  | | |  | | |  | EPQVYTLPPSRDELTKNQVSLTCLVK | | | | | | |  | | | 79 |  |  |
|  | | |  | | |  | FNWYVDGVEVHNAK | | | | | | |  | | | 93 |  |  |
|  | | |  | | |  | GFYPSDIAVEWESNGQPENNYKTTPPVLDSDGSFFLYSK | | | | | | |  | | | 82 |  |  |
|  | | |  | | |  | GPSVFPLAPSSK | | | | | | |  | | | 57 |  |  |
|  | | |  | | |  | GPSVFPLAPSSKSTSGGTAALGCLVK | | | | | | |  | | | 78 |  |  |
|  | | |  | | |  | GQPREPQVYTLPPSRDELTK | | | | | | |  | | | 62 |  |  |
|  | | |  | | |  | SCDKTHTCPPCPAPELLGGPSVFLFPPKPK | | | | | | |  | | | 68 |  |  |
|  | | |  | | |  | SCDKTHTCPPCPAPELLGGPSVFLFPPKPKDTLMISR | | | | | | |  | | | 50 |  |  |
|  | | |  | | |  | THTCPPCPAPELLGGPSVFLFPPKPK | | | | | | |  | | | 60 |  |  |
|  | | |  | | |  | TPEVTCVVVDVSHEDPEVK | | | | | | |  | | | 101 |  |  |
|  | | |  | | |  | TPEVTCVVVDVSHEDPEVKFNWYVDGVEVHNAK | | | | | | |  | | | 94 |  |  |
|  | | |  | | |  | TTPPVLDSDGSFFLYSK | | | | | | |  | | | 93 |  |  |
|  | | |  | | |  | TTPPVLDSDGSFFLYSKLTVDK | | | | | | |  | | | 69 |  |  |
|  | | |  | | |  | TTPPVLDSDGSFFLYSKLTVDKSR | | | | | | |  | | | 71 |  |  |
| IGHG3 | | | P01860 | | |  | CPAPELLGGPSVFLFPPKPK | | | | | | |  | | | 70 |  |  |
|  | | |  | | |  | CPAPELLGGPSVFLFPPKPKDTLMISR | | | | | | |  | | | 56 |  |  |
|  | | |  | | |  | RVELKTPLGDTTHTCPR | | | | | | |  | | | 80 |  |  |
|  | | |  | | |  | TPLGDTTHTCPR | | | | | | |  | | | 78 |  |  |
|  | | |  | | |  | VELKTPLGDTTHTCPR | | | | | | |  | | | 79 |  |  |
|  | | |  | | |  | TPEVTCVVVDVSHEDPEVQFK | | | | | | |  | | | 74 |  |  |
| IGHG2 | | | P01859 | | |  | KCCVECPPCPAPPVAGPSVFLFPPKPK | | | | | | |  | | | 51 |  |  |
|  | | |  | | |  | TPEVTCVVVDVSHEDPEVQFNWYVDGVEVHNAK | | | | | | |  | | | 81 |  |  |
|  | | |  | | |  | TTPPMLDSDGSFFLYSK | | | | | | |  | | | 99 |  |  |
|  | | |  | | |  | VVSVLTVVHQDWLNGK | | | | | | |  | | | 105 |  |  |
|  | | |  | | |  | VVSVLTVVHQDWLNGKEYK | | | | | | |  | | | 75 |  |  |
| LV302 | | | P80748 | | |  | FSGSNSGNTATLTISR | | | | | | |  | | | 108 |  |  |
|  | | |  | | |  | LTVLSQPK | | | | | | |  | | | 50 |  |  |
| KV119 | | | P01611 | | |  | DIQMTQSPSSVSASVGDR | | | | | | |  | | | 105 |  |  |
| HV304 | | | P01765 | | |  | AEDTAVYYCAK | | | | | | | HV303 (P01764) | | | 77 |  |  |
|  | | |  | | |  | EVQLLESGGGLVQPGGSLR | | | | | | | HV313 (P01774), HV315 (P01776), HV318 (P01779) | | | 118 |  |  |
| IGKC | | | P01834 | | |  | ADYEKHKVYACEVTHQGLSSPVTK | | | | |  | | | | | 72 |  |  |
|  | | |  | | |  | DSTYSLSSTLTLSK | | | | | | |  | | | 87 |  |  |
|  | | |  | | |  | HKVYACEVTHQGLSSPVTK | | | | | | |  | | | 124 |  |  |
|  | | |  | | |  | SGTASVVCLLNNFYPR | | | | | | |  | | | 98 |  |  |
|  | | |  | | |  | SGTASVVCLLNNFYPREAK | | | | | | |  | | | 60 |  |  |
|  | | |  | | |  | TVAAPSVFIFPPSDEQLK | | | | | | |  | | | 98 |  |  |
|  | | |  | | |  | TVAAPSVFIFPPSDEQLKSGTASVVCLLNNFYPR | | | | | | |  | | | 66 |  |  |
|  | | |  | | |  | TVAAPSVFIFPPSDEQLKSGTASVVCLLNNFYPREAK | | | | | | |  | | | 61 |  |  |
|  | | |  | | |  | VDNALQSGNSQESVTEQDSK | | | | | | |  | | | 139 |  |  |
|  | | |  | | |  | VDNALQSGNSQESVTEQDSKDSTYSLSSTLTLSK | | | | | | |  | | | 135 |  |  |
|  | | |  | | |  | VDNALQSGNSQESVTEQDSKDSTYSLSSTLTLSKADYEK | | | | | | |  | | | 123 |  |  |
|  | | |  | | |  | VQWKVDNALQSGNSQESVTEQDSK | | | | | | |  | | | 104 |  |  |
|  | | |  | | |  | VQWKVDNALQSGNSQESVTEQDSKDSTYSLSSTLTLSK | | | | | | |  | | | 127 |  |  |
|  | | |  | | |  | VYACEVTHQGLSSPVTK | | | | | | |  | | | 88 |  |  |
|  | | |  | | |  | VYACEVTHQGLSSPVTKSFNR | | | | | | |  | | | 81 |  |  |
|  | | |  | | |  | VYACEVTHQGLSSPVTKSFNRGEC | | | | | | |  | | | 69 |  |  |
| KV204 | | | P01617 | | |  | ASGVPDRFSGSGSGTDFTLK | | | | | | | KV201 (P01614), KV205 (P06309) | | | 86 |  |  |
|  | | |  | | |  | DIVMTQSPLSLPVTPGEPASISCR | | | | | |  | | | | 83 |  |  |
| LAC1/IGLL5 | | | P0CG04/B9A064 | | |  | ADGSPVKAGVETTKPSK | | | | | | |  | | | 75 |  |  |
|  | | |  | | |  | ADGSPVKAGVETTKPSKQSNNK | | | | | | |  | | | 76 |  |  |
|  | | |  | | |  | AGVETTKPSK | | | | | | |  | | | 68 |  |  |
|  | | |  | | |  | AGVETTKPSKQSNNK | | | | | | |  | | | 94 |  |  |
|  | | |  | | |  | AGVETTKPSKQSNNKYAASSYLSLTPEQWK | | | | | | |  | | | 68 |  |  |
|  | | |  | | |  | ANPTVTLFPPSSEELQANK | | | | | | |  | | | 95 |  |  |
| LV301 | | | P01714 | | |  | ITCQGDSLR | | | | | | |  | | | 55 |  |  |
|  | | |  | | |  | SELTQDPAVSVALGQTVR | | | | | | |  | | | 112 |  |  |
| HV107/HV106 | | | P06326/1761 | | |  | QVQLVQSGAEVK | | | | | | |  | | | 81 |  |  |
| HV301 | | | P01762 | | | | QVQLVQSGGGLVKPGGSLR | | | | | | |  | | | 92 |  |  |
| KV402 | | P01625 | | | |  | DIVMTQSPDSLAVSLGER | | | | | | |  | | | 104 |  |  |
| HV305 | | P01766 | | | |  | EVQLVESGGGLVQPGGSLR | | | | | | | HV316 (P01777) | | | 130 |  |  |
|  | |  | | | |  | AEDTAVYYCAR | | | | | | | HV302 (P01763), HV306 (P01767) | | | 73 |  |  |
| LAC3 | | P0CG06 | | | |  | SHKSYSCQVTHEGSTVEK | | | | | |  | | | | 96 |  |  |
|  | |  | | | |  | SHKSYSCQVTHEGSTVEKTVAPTECS | | | | | | |  | | | 70 |  |  |
| KV106 | | P01598 | | | |  | ASSLESGVPSR | | | | | |  | | | | 91 |  |  |
|  | |  | | | |  | DIQMTQSPSTLSASVGDR | | | | | | | KV104 (P01596) | | | 112 |  |  |
| LV102 | | P01700 | | | |  | RPSGVPDRFSGSK | | | | | | | LV206 (P01709), LV209 (P01712) | | | 66 |  |  |
|  | |  | | | |  | SGTSASLAISGLR | | | | | | LV106 (P04208) | | | | 84 |  |  |
| HV320 | | P01781 | | | |  | GLEWVANIK | | | | | | |  | | | 68 |  |  |
|  | |  | | | |  | NSLYLQMNSLR | | | | | |  | | | | 72 |  |  |
|  | |  | | | |  | VEDTALYYCAR | | | | | | |  | | | 86 |  |  |
| KV101 | | P01593 | | | |  | ILIYDASNLETGVPSR | | | | | | |  | | | 100 |  |  |
|  | |  | | | |  | TFGQGTKLEIK | | | | | | |  | | | 71 |  |  |
|  | |  | | | |  | TFGQGTKLEIKR | | | | | | |  | | | 70 |  |  |
| KV305 | | P01623 | | | |  | ATGIPDRFSGSGSGTDFTLTISR | | | | KV302 (P01620), KV304 (P01622), KV307(P04206), KV312 (P18135), KV313 (P18136) | | | | | | 82 |  |  |
|  | |  | | | |  | EIVLTQSPGTLSLSPGER | | KV302 (P01620), KV304 (P01622), KV307(P04206), | | | | | | | | 98 |  |  |
|  | |  | | | |  | FSGSGSGTDFTLTISR | | KV302 (P01620), KV304 (P01622), KV312 (P18135), KV313 (P18136) | | | | | | | | 117 |  |  |
|  | |  | | | |  | TFGQGTKVEIKR | KV312 (P18135) | | | | | | | | | 74 |  |  |
| HV308 | | P01769 | | | |  | AENTAVYYCAR | | |  | | | | | | | 75 |  |  |
|  | |  | | | |  | QVQLVQSGGGAVQPGRSLR | | |  | | | | | | | 66 |  |  |
| LV403 | | P01717 | | | |  | SYELTQPPSVSVSPGQTAR | | | | | | | | |  | 97 |  |  |
| HV209/HV207 | | P06331/1825 | | | |  | LSSVTAADTAVYYCAR | | | | | | |  | | | 101 |  |  |
